# Lack of PINK1 alters glia innate immune responses and enhances inflammation-induced, nitric oxide-mediated neuron death

**DOI:** 10.1038/s41598-017-18786-w

**Published:** 2018-01-10

**Authors:** Liuke Sun, Ruifang Shen, Sandeep K. Agnihotri, Yun Chen, Zhiwei Huang, Hansruedi Büeler

**Affiliations:** HIT Center for Life Sciences, and School of Life Sciences and Technology, Harbin Institute of Technology, Harbin, 150080 China

## Abstract

Neuroinflammation is involved in the pathogenesis of Parkinson’s disease (PD) and other neurodegenerative disorders. We show that lack of PINK1- a mitochondrial kinase linked to recessive familial PD – leads to glia type-specific abnormalities of innate immunity. PINK1 loss enhances LPS/IFN-γ stimulated pro-inflammatory phenotypes of mixed astrocytes/microglia (increased iNOS, nitric oxide and COX-2, reduced IL-10) and pure astrocytes (increased iNOS, nitric oxide, TNF-α and IL-1β), while attenuating expression of both pro-inflammatory (TNF-α, IL-1β) and anti-inflammatory (IL-10) cytokines in microglia. These abnormalities are associated with increased inflammation-induced NF-κB signaling in astrocytes, and cause enhanced death of neurons co-cultured with inflamed *PINK1* ^−/−^ mixed glia and neuroblastoma cells exposed to conditioned medium from LPS/IFN-γ treated *PINK1* ^−/−^ mixed glia. Neuroblastoma cell death is prevented with an iNOS inhibitor, implicating increased nitric oxide production as the cause for enhanced death. Finally, we show for the first time that lack of a recessive PD gene (PINK1) increases α-Synuclein-induced nitric oxide production in all glia types (mixed glia, astrocytes and microglia). Our results describe a novel pathogenic mechanism in recessive PD, where PINK1 deficiency may increase neuron death via exacerbation of inflammatory stimuli-induced nitric oxide production and abnormal innate immune responses in glia cells.

## Introduction

Neuroinflammation plays an important role in the pathogenesis of PD^1,2^. Brain inflammation contributes to the neuropathology in toxin-induced models of PD^2^. In addition, the dominant PD-linked genes α-Synuclein (αS)^3–7^ and LRRK2^3,8–11^ promote neuroinflammation through activation of microglia and specific inflammatory signaling pathways. αS activates both microglia and astrocytes via Toll-like receptors (TLRs)^12–15^, leading to excessive production of pro-inflammatory cytokines and nitric oxide (NO)^12,13,16^. In contrast, relatively few studies have addressed the importance of recessive PD genes in neuroinflammation, although expression of PINK1, Parkin and DJ-1 is increased in reactive astrocytes in the diseased human brain^17–19^, suggesting that these proteins affect or regulate glia-dependent immune responses. Parkin was shown to suppress LPS-induced expression of iNOS, IL-1β and TNF-α in peripheral macrophages, and Parkin itself may be a target for inflammation-induced inactivation via NF-κB-dependent suppression of Parkin gene transcription^20^. In addition, Parkin-deficient mice were slightly more vulnerable to chronic LPS-induced dopamine neuron loss, although brain inflammation was comparable to wildtype mice^21^. Loss of DJ-1 strongly increased NO production and expression of IL-6 and COX-2 by LPS-treated astrocytes, which was mediated by increased phosphorylation and activity of p38MAPK and correlated with reduced survival of co-cultured neurons, compared to wildtype astrocytes^22^. PINK1 is a mitochondrial kinase linked to recessive familial PD^23^ and regulates mitochondrial quality control via its ability to promote mitophagy of depolarized mitochondria^24^. In cells and animals, PINK1 deficiency is associated with increased levels of reactive oxygen species^25–29^, heightened oxidative stress sensitivity^30^ as well as abnormal mitochondrial function^25,29–31^ and morphology^31,32^. However, the mitochondrial phenotypes of PINK1 loss may vary considerably among different cell types depending on cell metabolism and cell type-specific adaptations^31,33^. Only few studies have investigated the importance of PINK1 in neuroinflammation and inflammation-related signal transduction. Increased p38MAPK activation was reported in PINK1-deficient astrocytes^29^, and loss of PINK1 enhanced the release of inflammatory cytokines from acutely prepared brain slices mimicking brain injury^34^, suggesting an anti-inflammatory function of PINK1. On the other hand, PINK1 was shown to stimulate pro-inflammatory IL-1β signal transduction via interaction with several proteins that function downstream of the IL-1β receptor^35,36^. Currently, a detailed characterization of the effects of PINK1 loss on inflammatory gene expression in glia and the ability of *PINK1* ^−/−^ glia to protect neurons against inflammation-induced death are missing. Here we show that lack of PINK1 differentially affects inflammation-induced gene expression and NO production in astrocytes, microglia and mixed astrocytes/microglia. These changes are associated with increased apoptosis of normal primary WT neurons co-cultured with PINK1-deficient mixed glia. Moreover, SH-SY5Y neuroblastoma cells exposed to conditioned medium of PINK1-deficient mixed glia show increased death that can be prevented with an inhibitor of iNOS. Finally, we show that PINK1 loss also enhances αS-stimulated NO production by glia. Overall, our results suggest that PINK1 deficiency in recessive familial Parkinsonism may increase neuron death via exacerbation of inflammatory stimuli-induced NO production and abnormal innate immune responses in glia cells.

## Results

### Characterization of primary mixed glia

**Figure 1:**
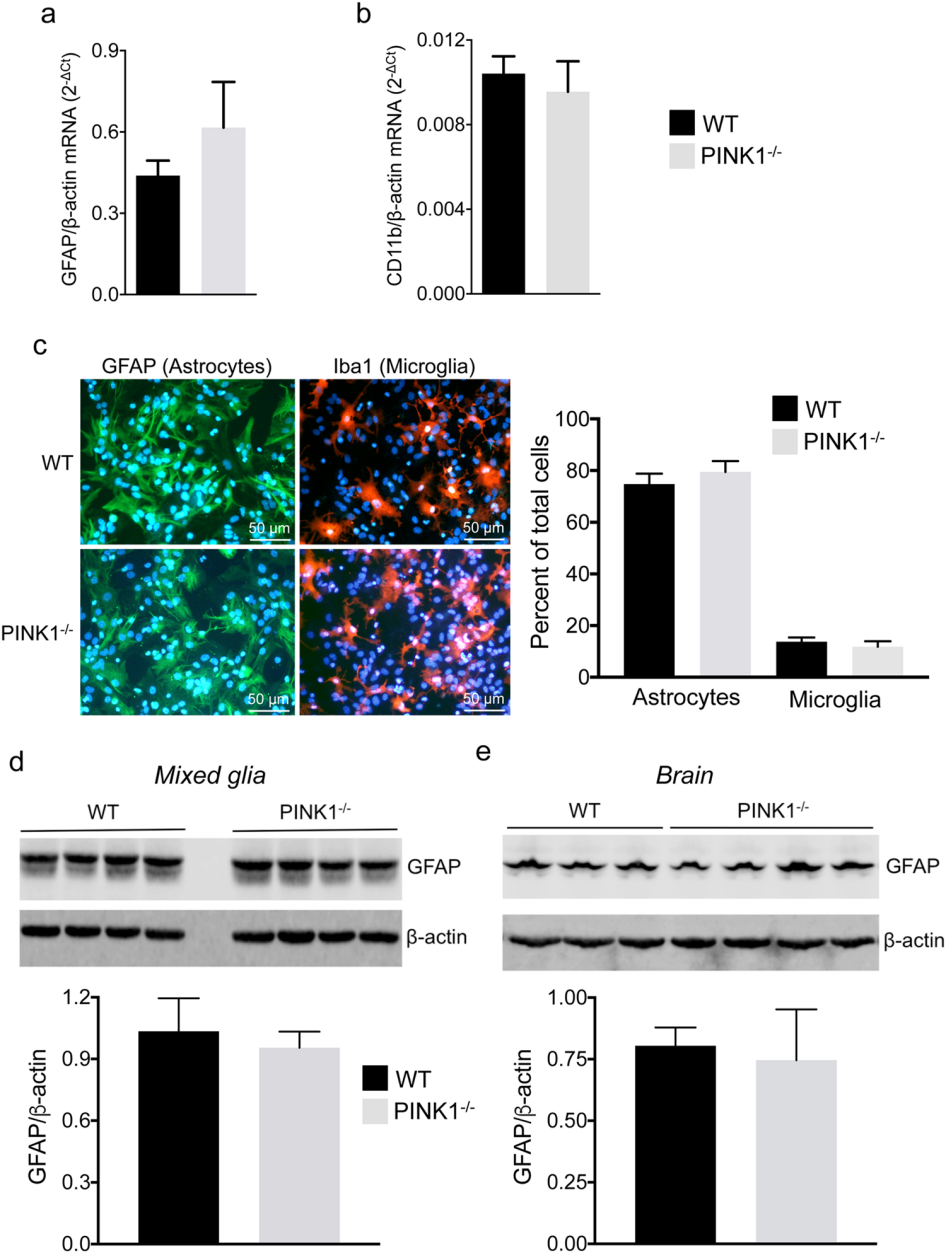
Gene expression analysis and cell type composition of primary mixed glia of WT and *PINK1* ^−/−^ mice. Expression of mRNAs for (**a**) the astrocyte marker GFAP and (**b**) the microglia marker CD11b in primary mixed glia cultures from the cortex of neonatal mice (n = 4 mice/genotype). Expression is shown as 2^−ΔCt^ with β-actin as the internal reference gene. (**c**) Percentage of astrocytes and microglia in mixed glia cultures. Astrocytes and microglia were identified in separate cultures by immunocytochemistry with antibodies against GFAP and Iba-1. Total number of cells were determined by nuclear DAPI stain. N = 4 mice/genotype were analyzed, and 1368–2118 total cells were counted per mouse. (**d**) Expression of GFAP protein in WT and *PINK1* ^−/−^ primary mixed neocortical cells (n = 4 mice/genotype). (**e**) Expression of GFAP protein in whole brain of n = 3 WT and n = 4 *PINK1* ^−/−^ mice. Blots in Fig. 1d and Fig. 1e were cropped and full-length blots are shown in Supplementary Fig. 6.

To characterize the cell composition and purity of mixed astrocyte/microglia cultures, astrocytes and microglia, we carried out quantitative real-time PCR and immunocytochemistry for glia-type specific genes and proteins, respectively. Expression of GFAP mRNA **(**Fig. 1a **)** and CD11b mRNA **(**Fig. 1b **)** was comparable in WT and PINK1-deficient mixed glia cultures. In addition, the percentage of astrocytes and microglia was comparable in WT and *PINK1* ^−/−^ mixed glia as determined by immunocytochemistry and cell counting (Fig. 1c). Finally, the amount of GFAP protein in *PINK1* ^−/−^ mixed cultures **(**Fig. 1d **)** and in the brain of *PINK1* ^−/−^ mice **(**Fig. 1e **)** was similar to WT samples. In summary, most of the cells in the mixed cultures are astrocytes and 12–14% are microglia. There is no difference in the proportions of the cell types, and in the expression of cell-type specific markers, between mixed glia cultures from WT and *PINK1* ^−/−^ mice.

### Increased NO production and altered inflammatory gene expression in LPS/IFN-γ treated PINK1-deficient mixed astrocyte/microglia cultures

**Figure 2:**
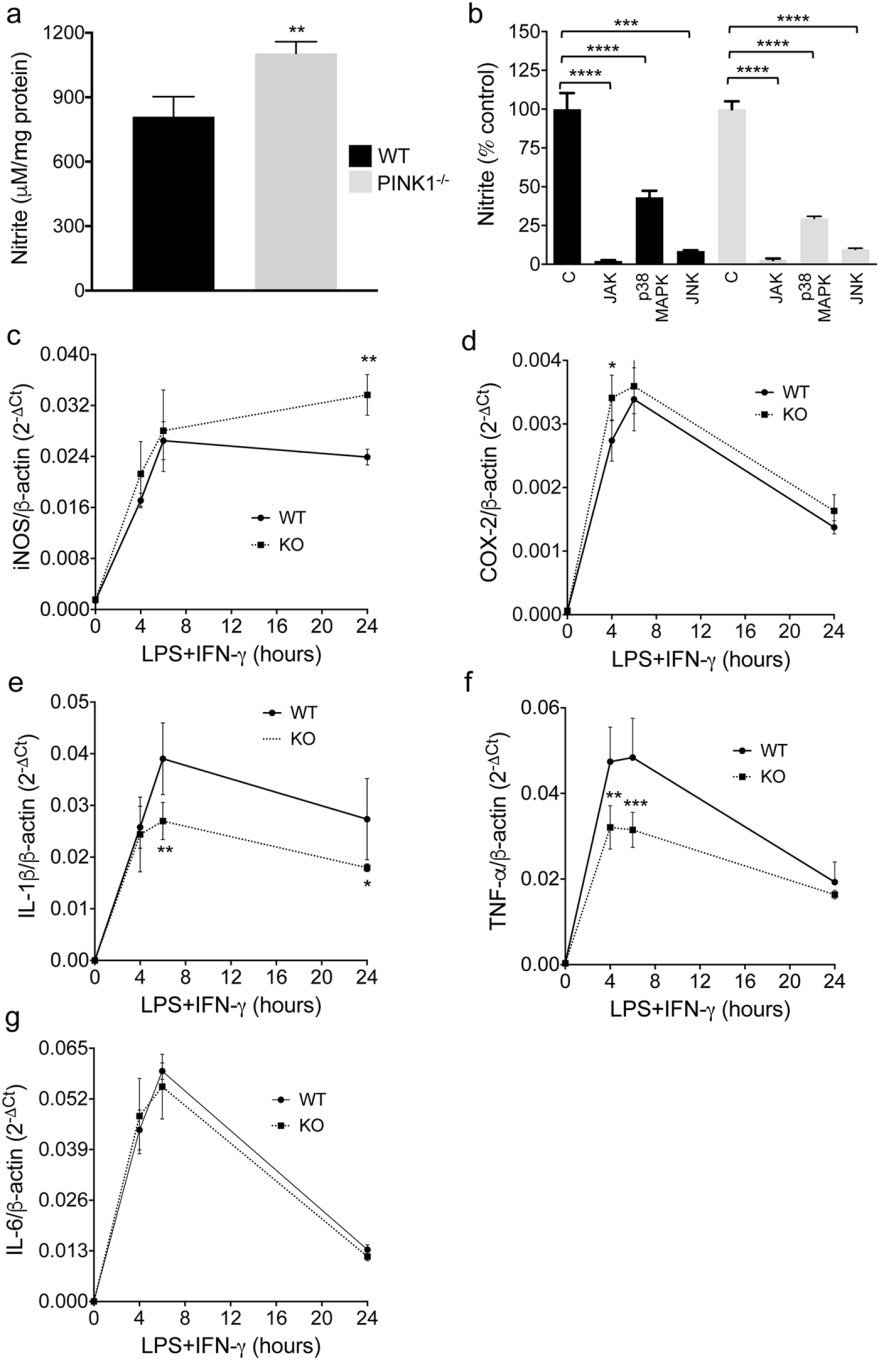
PINK1 loss increases NO production and alters expression of inflammatory enzymes and cytokines in LPS/IFN-γ stimulated mixed glia. (**a**) NO levels, measured as nitrite in the Griess assay, are increased in PINK1-deficient cultures 24 hours after LPS/IFN-γ treatment. N = 3 mice/per genotype. (**b**) NO production in presence of specific inhibitors of Janus kinases (GLPG0634, 30 μM), p38MAPK (SB203580, 30 μM) and JNKs (SP600125, 20 μM). N = 4 mice/genotype. (**c**–**g**) Quantification of mRNA expression by real-time PCR, showing increased expression of iNOS and COX-2, decreased expression of IL-1β and TNF-α and unaltered expression of IL-6 at various time points of LPS/IFN-γ stimulation. N = 4 mice/genotype. All data are mean ± SD. *p < 0.05, **p < 0.01, ***p < 0.001, ****p < 0.0001.

Increased expression of iNOS and COX-2 has been observed in the brain of PD patients and is causally linked to neurodegeneration in models of PD^11,37–39^. In mixed glia, NO was undetectable in both genotypes under control conditions. In contrast, twenty-four hours after treatment with LPS and IFN-γ to mimic inflammation, NO levels were significantly higher in *PINK1* ^−/−^ cultures compared to WT **(**Fig. 2a **)**. To study the importance of various signal transduction pathways for NO production, LPS/IFN-γ stimulation of mixed glia was performed in the presence of specific inhibitors of Janus kinases (JAKs), p38 mitogen-activated protein kinase (p38MAPK) or Jun N-terminal kinases (JNKs). All inhibitors led to significant (partial or complete) reduction of NO production in both WT and *PINK1* ^−/−^ mixed glia. In addition, the relative reduction of NO production for all inhibitors was similar for the two genotypes **(**Fig. 2b **)**. Consistent with increased NO production, mRNA levels for iNOS were higher in mixed glia lacking PINK1 compared to WT twenty-four hours after LPS/IFN-γ treatment **(**Fig. 2c **)**. In addition, COX-2 expression was increased in *PINK1* ^−/−^ mixed glia four hours after LPS/IFN-γ stimulation **(**Fig. 2d **)**. The transcript levels for IL-1β (at six and twenty-four hours) and for TNF-α (at four and six hours) were lower in LPS/IFN-γ stimulated *PINK1* ^−/−^ mixed glia compared to WT cells **(**Fig. 2e,f **)**, whereas no difference in the expression of IL-6 was observed in the two genotypes **(**Fig. 2g **)**. In summary, these results show that loss of PINK1 increases NO production and affects the expression of inflammatory genes in LPS/IFN-γ stimulated mixed astrocyte/microglia cultures in complex ways, increasing some (iNOS, COX-2) while decreasing others (IL-1β, TNF-α).

### PINK1 loss differentially affects pro-inflammatory gene expression and NO production in astrocytes and microglia

**Figure 3:**
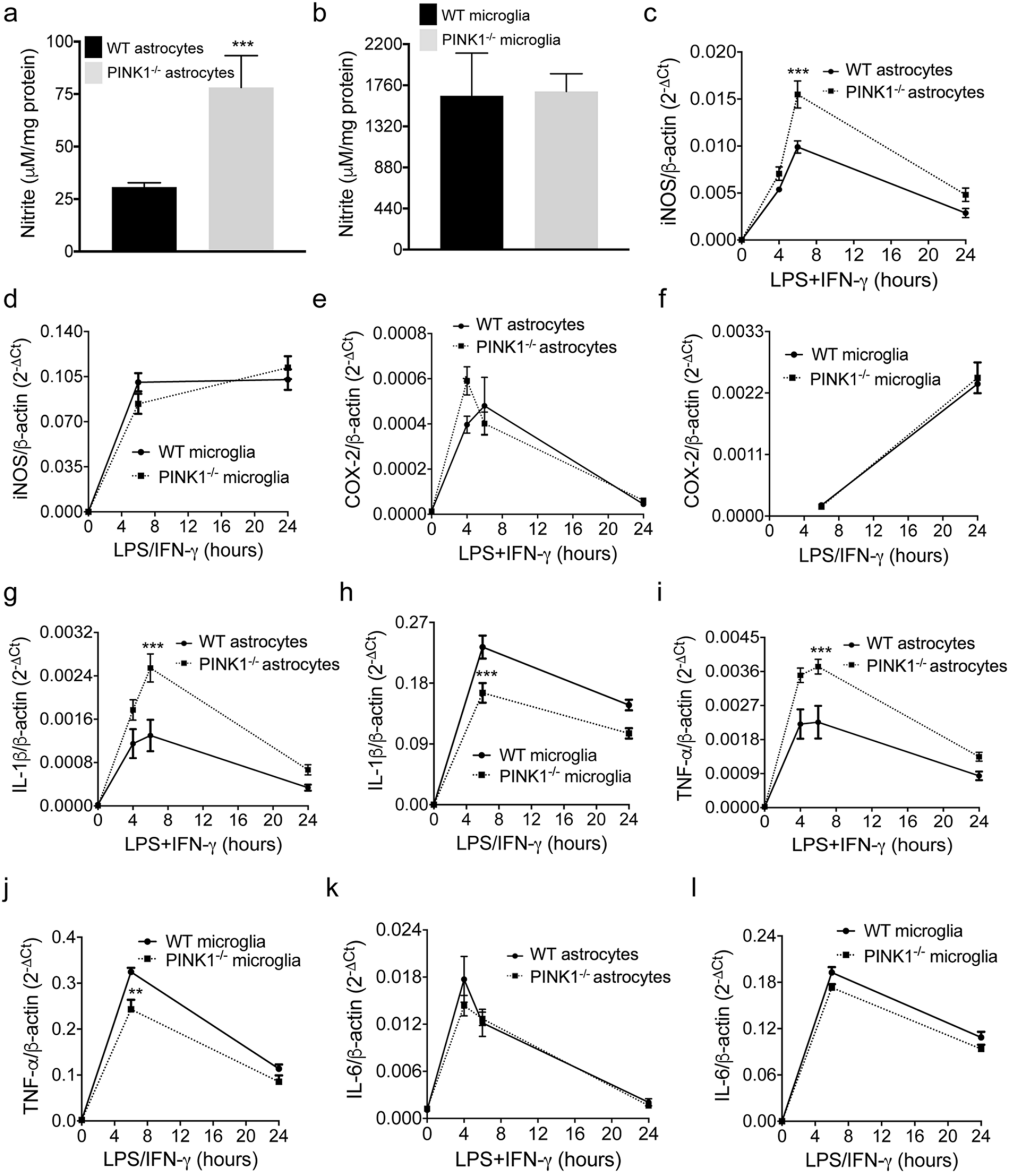
NO production and inflammatory gene expression in pure astrocytes and microglia. (**a**–**b**) NO-derived nitrite in the medium of LPS/IFN-γ stimulated WT and *PINK1* ^−/−^ (**a**) astrocytes and (**b**) microglia (n = 4 mice/genotype, mean ± SD). (**c**–**l**) Real-time PCR quantification of mRNA levels for pro-inflammatory genes in astrocytes (**c**,**e**,**g**,**i**,**k**) and microglia (**d**,**f**,**h**,**j**,**l**) (n = 4 mice/genotype, mean ± SEM). *p < 0.05, **p < 0.01, ***p < 0.001.

To gain a better understanding of the mechanisms underlying altered neuroinflammation in mixed glia, we carried out analogous experiments with pure microglia and astrocytes. Characterization of the purity of astrocytes and microglia cultures is shown in Supplementary Fig. 1, and in both cases was > 99% with no difference between genotypes. Upon treatment with LPS/IFN-γ, lack of PINK1 resulted in higher NO production **(**Fig. 3a **)** and iNOS expression **(**Fig. 3c **)** in astrocytes, while in *PINK1* ^−/−^ microglia NO levels **(**Fig. 3b **)** and iNOS expression **(**Fig. 3d **)** were comparable to WT. COX-2 transcript levels were normal in both *PINK1* ^−/−^ astrocytes and microglia **(**Fig. 3e,f **)**. Interestingly, expression of IL-1β **(**Fig. 3g **)** and TNF-α **(**Fig. 3i **)** were increased in *PINK1* ^−/−^ astrocytes, whereas the same cytokines showed reduced expression in PINK1-deficient microglia **(**Fig. 3h,j **)**. Finally, no change in IL-6 expression was found in astrocytes or microglia lacking PINK1 **(**Fig. 3k,l **)**. In summary, PINK1 deficiency increases the levels of pro-inflammatory mediators in LPS/IFN-γ treated astrocytes but reduces pro-inflammatory cytokine expression in microglia.

### PINK1 deficiency leads to lower IL-10 expression in microglia and increased TGF-β1 expression in astrocytes

**Figure 4:**
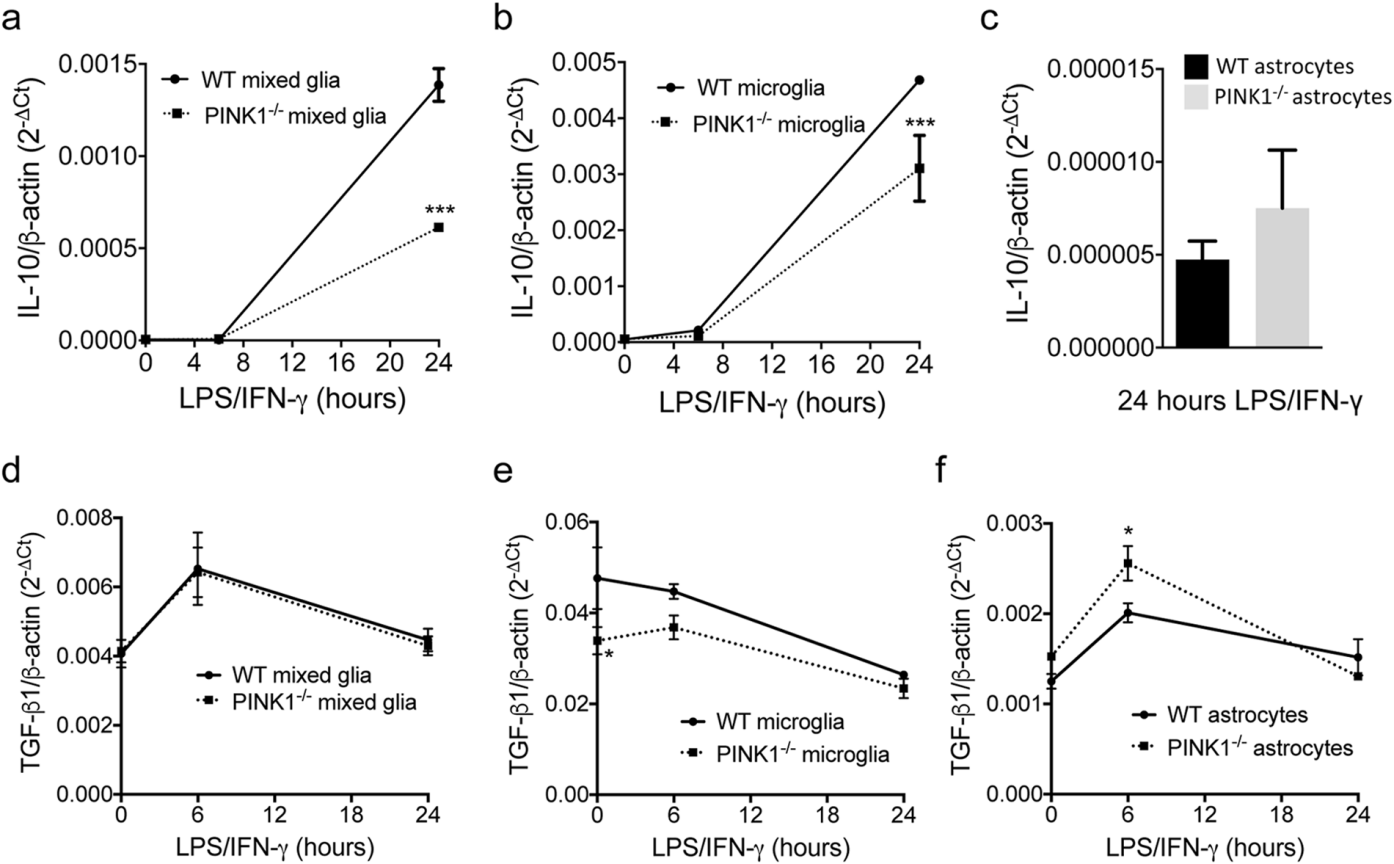
IL-10 and TGF-β1 expression in mixed glia, astrocytes and microglia. Expression of IL-10 (**a**–**c**) and TGF-β1 (**d**–**f**) in LPS/IFN-γ stimulated WT and *PINK1* ^−/−^ mixed glia (**a**,**d**), microglia (**b**,**e**) and astrocytes (**c**,**f**). N = 4 mice/genotype, mean ± SEM. *p < 0.05, ***p < 0.001.

IL-10 is a potent anti-inflammatory cytokine produced by microglia that inhibits production of TNF-α, decreases NO biosynthesis and attenuates immunological activation of astrocytes^40^. In addition, TGF-β is a modulator of brain inflammation that may inhibit^41,42^ or promote^43–45^ neuroinflammation, depending on context and disease. We show that IL-10 expression is significantly lower in both mixed glia **(**Fig. 4a **)** and pure microglia **(**Fig. 4b **)** lacking PINK1 twenty-four hours after LPS/IFN-γ stimulation, while virtually no IL-10 was made by LPS/IFN-γ stimulated astrocytes of either genotype **(**Fig. 4c **)**. There was no difference in TGF-β mRNA expression in mixed glia **(**Fig. 4d **)**. However, *PINK1* ^−/−^ microglia expressed lower levels of TGF-β1 under basal conditions **(**Fig. 4e **)**, while TGF-β expression was higher in *PINK1* ^−/−^ astrocytes six hours after LPS/IFN-γ incubation **(**Fig. 4f **)**.

### PINK1 loss increases αS-induced NO production in glia

**Figure 5:**
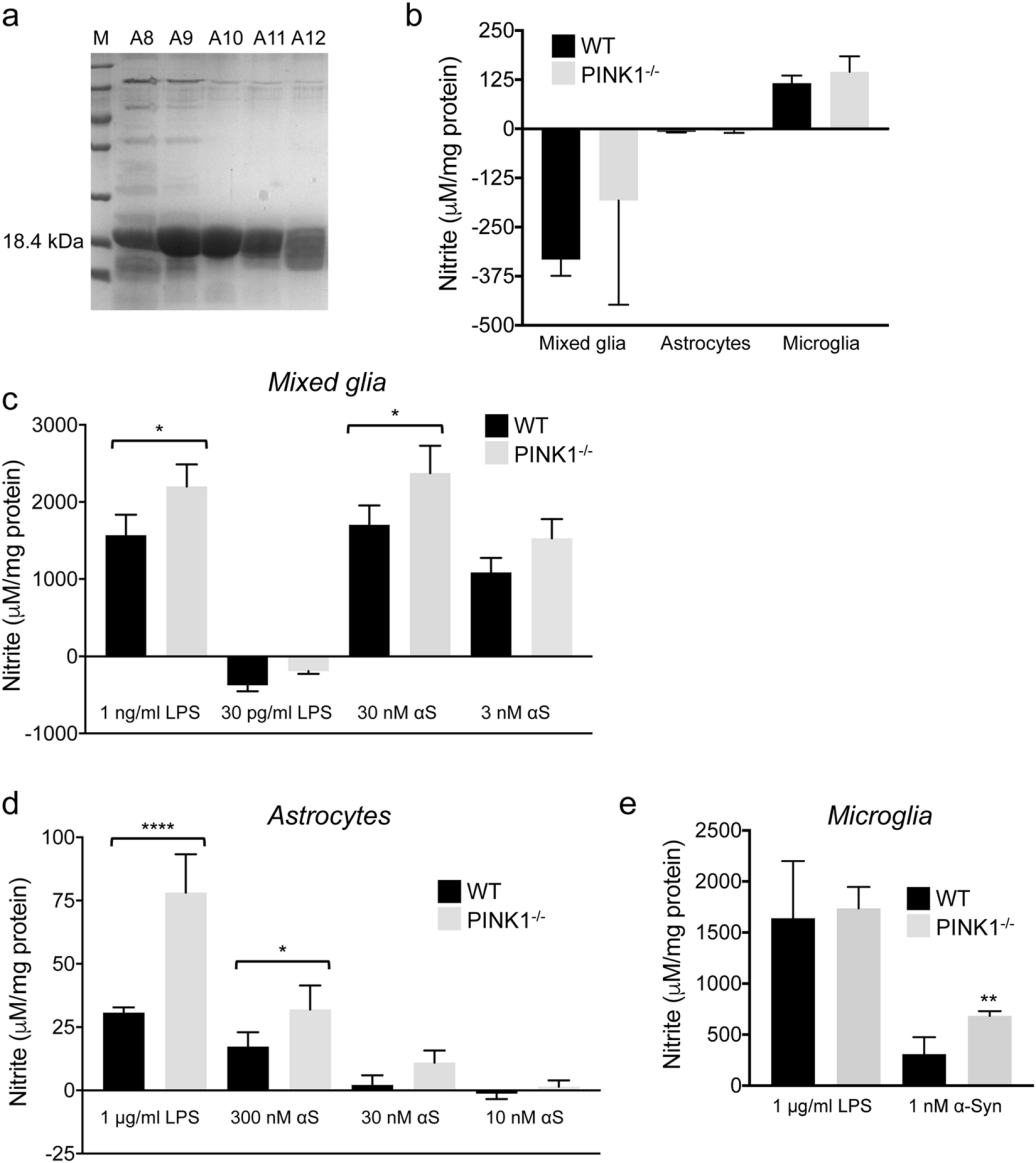
Lack of PINK1 increases α-Synuclein-induced NO production in glia. (**a**) SDS polyacrylamide gel showing fractions of recombinant mouse αS eluted from the Resource Q column. Fractions A10 and A11 were used for experiments (without prior incubation and/or shaking). (**b**) NO-derived nitrite in supernatants of mixed glia, astrocytes and microglia incubated with IFN-γ alone (n = 4 mice/genotype and cell type). (**c**-**e**) Nitrite in supernatants of mixed glia (**c**), astrocytes (**d**) and microglia (**e**) incubated with various concentrations of αS or LPS in presence of 10 ng/ml IFN-γ (n = 4 mice/genotype and cell type). Nitrite was measured with the Griess assay and normalized to protein content in the cell extract. Mean ± SD, *p < 0.05, **p < 0.01, ****p < 0.0001.

Recombinant αS was expressed in *E*. *coli*, and αS purified from bacteria is shown in Fig. 5a. We used pooled fractions A10 and A11 for experiments, without prior incubation to form αS aggregates. Because LPS itself can result in NO production, the amount of LPS present in the αS preparation was measured with a competitive ELISA. This showed that one microgram of purified αS contained 7.3–15.1 pg of LPS **(**Supplementary Fig. 2 **)**. With a molecular mass of 14.5 kDa for αS, 30 nM αS contains 3.2–6.6 pg/ml LPS **(**Supplementary Fig. 2 **)**. Because it has recently been shown that PINK1-deficient mice are more vulnerable to αS-induced neuropathology and dopamine neuron loss^46,47^, we studied whether lack of PINK1 affects αS-induced neuroinflammation by measuring αS-induced NO production in different types of glia. In addition, we included 10 ng/ml IFN-γ in all experiments to compare results with NO production triggered by LPS and IFN-γ. Levels of NO were undetectable in mixed glia and astrocytes treated with IFN-γ alone, while low amounts of NO were produced in IFN-γ treated microglia **(**Fig. 5b **)**. 30 nM αS resulted in increased NO production in *PINK1* ^−/−^ mixed glia compared to WT cells **(**Fig. 5c **)**. In astrocytes, NO production with 300 nM (but not 30 nM) αS was also significantly higher in cells lacking PINK1 **(**Fig. 5d **)**. Microglia were more effective at producing NO, and 1 nM αS resulted in higher amounts of NO in *PINK1* ^−/−^ microglia compared to WT cells **(**Fig. 5e **)**. Because 30 pg/ml LPS failed to produce NO production (see Fig. 5c), we conclude that PINK1 deficiency increases αS-dependent NO production in mixed glia and pure microglia. On the other hand, because 300 nM αS (containing > 30 pg/ml LPS) was required to produce more NO in *PINK1* ^−/−^ astrocytes, we cannot currently conclude nor exclude that PINK1 loss also increased αS-dependent NO production in astrocytes.

### Lack of PINK1 increases mixed glia-mediated primary neuron apoptosis and NO-dependent neuroblastoma cell death

**Figure 6:**
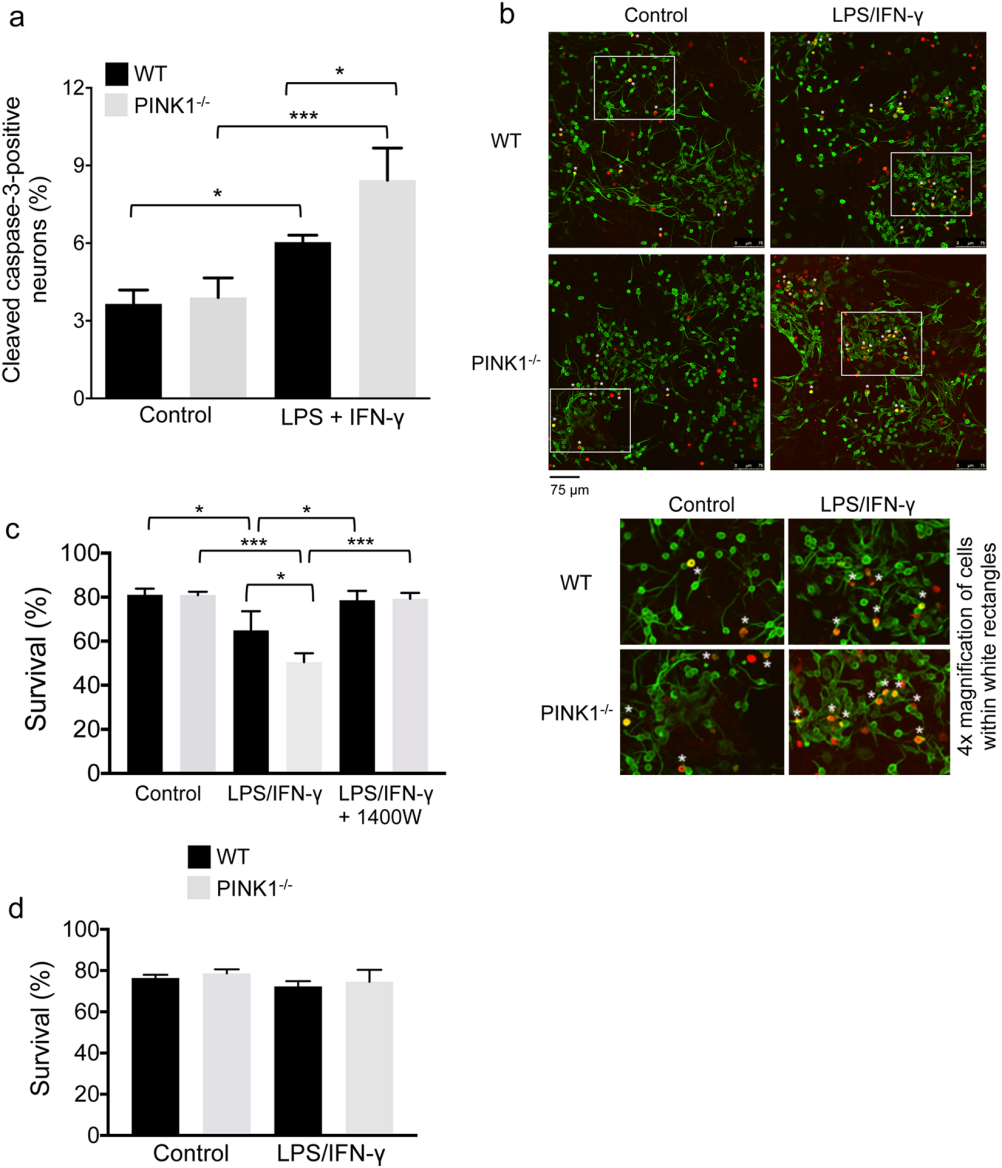
Lack of PINK1 increases mixed glia-mediated primary neuron apoptosis and NO-dependent SH-SY5Y cell death. (**a**–**b**) Apoptosis of primary cortical neurons from WT mice in co-cultures with WT or *PINK1* ^−/−^ mixed glia cells was measured by quantification of neurons (tubulin-βIII) that stained positive for active caspase-3. Without LPS/IFN-γ (control), neuronal apoptosis was unaltered by PINK1 deficiency. 24 h after treatment with LPS/IFN-γ, WT neurons grown on *PINK1* ^−/−^ mixed glia cells showed heightened apoptosis compared to WT neurons in co-cultures with WT mixed glia. Examples of apoptotic neurons are indicated with an asterisk in the overlays of panel b (cells within white rectangles are shown magnified at the bottom of panel b). N = 3 mice/genotype, 921–1787 total neurons were analyzed in three random view fields (images) per mouse (**c**) Survival of SH-SY5Y neuroblastoma cells after 48 hours of exposure to different types of conditioned medium (indicated below bars) collected from WT or *PINK1* ^−/−^ mixed glia. N = 3 mice/genotype. (**d**) Survival of SH-SY5Y neuroblastoma cells after 48 hours of exposure to different types of conditioned medium (indicated below bars) collected from WT or *PINK1* ^−/−^ astrocytes. N = 3 mice/genotype. Data show mean ± SD, *p < 0.05, ***p < 0.001.

Glia-derived NO is a diffusible molecule that can damage and kill neurons through inhibition of mitochondrial respiration, induction of mitochondrial permeability transition and protein oxidation/nitration^4,48,49^. To study whether PINK1 deficiency in glia cells increases apoptosis of neurons, primary cortical neurons from WT mice were plated onto confluent layers of mixed astrocytes/microglia from WT or *PINK1* ^−/−^ mice. The co-cultures were left untreated (control) or incubated for twenty-four hours with LPS/IFN-γ, and the percentage of WT neurons (tubulin-βIII^+^ cells) with active caspase-3 was quantified. In the absence of inflammation (LPS/IFN-γ), the apoptotic rate of WT neurons was the same whether grown on WT or *PINK1* ^−/−^ mixed glia cells **(**Fig. 6a,b **)**. As expected, treatment with LPS and IFN-γ led to an increase in apoptosis of neurons grown on mixed glia of both genotypes. However, apoptosis of neurons was significantly higher in co-cultures with *PINK1* ^−/−^ compared to WT mixed glia cells **(**Fig. 6a,b **)**. Because the neurons expressed functional PINK1 in both types of co-cultures, these results show that loss of PINK1 in glia cells is sufficient to increase apoptosis of co-cultured neurons under inflammatory conditions. Based on the increased pro-inflammatory phenotypes of *PINK1* ^−/−^ mixed glia and astrocytes we further measured death of SH-SY5Y neuroblastoma cells exposed to conditioned medium from these two types of glia. In addition, we tested whether pharmacological iNOS inhibition blocked inflammation-induced SH-SY5Y cell death. Conditioned medium from LPS/IFN-γ treated mixed glia of both genotypes reduced the survival of SH-SY5Y neuroblastoma cells **(**Fig. 6c **)**. However, survival of SH-SY5Y cells exposed to *PINK1* ^−/−^ conditioned medium was significantly lower than that of SH-SY5Y cells exposed to WT conditioned medium **(**Fig. 6c **)**. The iNOS inhibitor 1400 W prevented LPS/IFN-γ induced SH-SY5Y cell death and restored survival to levels comparable to cells incubated with conditioned medium from control mixed glia **(**Fig. 6c **)**. In contrast, conditioned medium from LPS/IFN-γ treated astrocytes did not cause measurable SH-SY5Y cell death **(**Fig. 6d **)**, presumably because NO levels are significantly lower than in mixed glia and therefore insufficient to trigger neuron death. Collectively, these results show that PINK1 loss in primary mixed glia -which most closely resemble the situation in the brain-leads to increased LPS/IFN-γ induced primary neuron apoptosis, as well as increased neuroblastoma cell death that can be fully prevented with an iNOS inhibitor and is thus mediated by increased NO production.

### PINK1 loss augments NF-κB signaling in LPS/IFN-γ treated astrocytes

**Figure 7:**
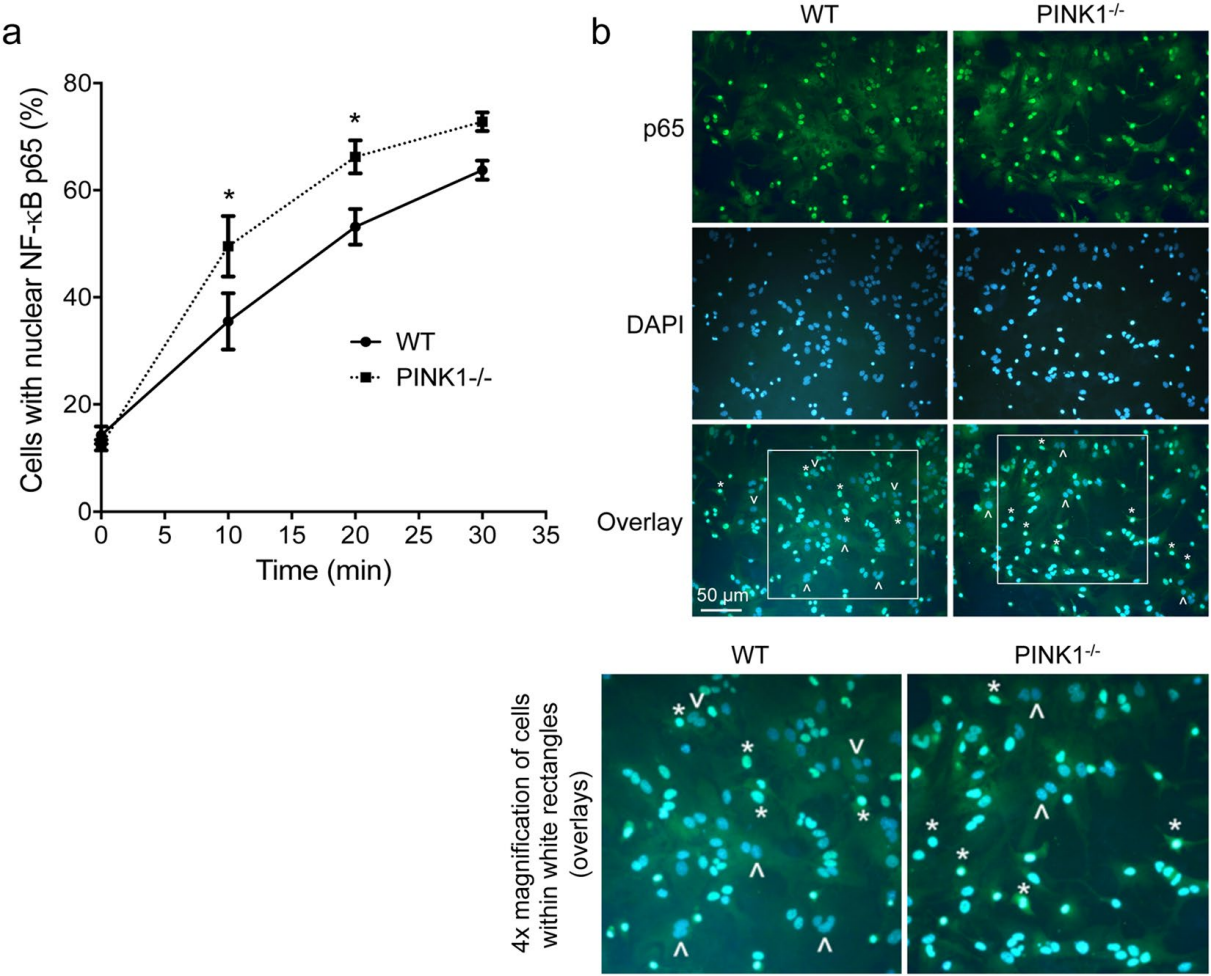
Astrocytes devoid of PINK1 display increased NF-κB activity. (**a**) Percentage of cells with NF-κB p65 subunit localized in the nucleus. (**b**) Representative images of cells with p65 in nucleus, at 20 min after LPS/IFN-γ treatment. The p65 subunit of NF-κB was detected with anti-p65 antibody and Alexa488-conjugated (green) secondary antibody. Nuclei were stained with DAPI (blue). In the overlays examples of cells with nuclear p65 (active NF-κB) are marked with an asterisk, while cells without nuclear p65 are indicated with an arrowhead (cells within white rectangles are shown magnified at the bottom of panel b). N = 4 mice/genotype, 441–925 total cells (nuclei) were analyzed in three random view fields (images) per mouse. Mean ± SEM, *p < 0.05 at 10 min and 20 min after LPS/IFN-γ.

The transcription factor NF-κB is required for the induction of the iNOS gene in response to LPS, cytokines and other compounds^50^. We studied NF-κB pathway activity by measuring nuclear translocation of the NF-κB p65 subunit in LPS/IFN-γ treated mixed glia. In the absence of LPS/IFN-γ (t = 0 min), the percentage of cells with p65 in the nucleus was the same for WT and *PINK1* ^−/−^ cells (WT: 14.3 ± 3.2%, *PINK1* ^−/−^: 12.4 ± 2.0%, p = 0.37). Upon stimulation with LPS/IFN-γ, p65 rapidly accumulated in the nucleus of cells of both genotypes, but the percentage of cells with nuclear p65 was significantly higher in *PINK1* ^−/−^ cells at both 10 min and 20 min after treatment **(**Fig. 7 **)**. Because the majority of the cells in the mixed glia cultures were astrocytes (75–79%, see Fig. 1c), this result demonstrates that loss of PINK1 increases inflammation-induced NF-κB signaling in astrocytes. Further supporting this notion, we show that iNOS mRNA levels are significantly higher in pure *PINK1* ^−/−^ astrocytes than in WT astrocytes at six hours after LPS/IFN-γ treatment **(**Fig. 3c **)**, and that NO levels remain higher in *PINK1* ^−/−^ astrocytes even twenty-four hours after inflammation **(**Fig. 3a **)**.

### Loss of PINK1 increases cytoplasmic and mitochondrial ROS levels in primary astrocytes

Astrocytes regulate synaptic neurotransmission and protect neurons by taking up glutamate, controlling extracellular potassium ion concentration, and releasing several neuroactive substances^51^. Because oxidative stress may affect astrocyte function, we measured the levels of ROS in normal, non-inflamed astrocytes. WT and PINK1-deficient primary astrocytes were transduced with lentiviral vectors expressing Cyto-roGFP and Matrix-roGFP, which are targeted to the cytoplasm and mitochondria of cells and show ROS-dependent ratiometric fluorescence when excited at two different wavelengths (405 nm and 488 nm). The F405/F488 (fluorescence) ratio was increased in both cytoplasm and mitochondrial matrix of *PINK1* ^−/−^ primary astrocytes compared to WT cells **(**Supplementary Fig. 3 **)**. Therefore, loss of PINK1 leads to heightened levels of ROS in the cytoplasm and mitochondria of astrocytes under basal conditions. In addition, we examined whether treatment of astrocytes with LPS and IFN-γ further exacerbated ROS in the mitochondrial matrix, but found that this was not the case in either genotype **(**Supplementary Fig. 3 **)**.

### PINK1-deficient primary astrocytes display impaired mitochondrial function under basal conditions

To study whether loss of PINK1 resulted in altered mitochondrial dynamics in astrocytes, we infected primary astrocytes with a retrovirus that expresses mitochondria-targeted roGFP (Matrix-roGFP). No gross abnormalities in mitochondrial morphology were observed in PINK1-deficient astrocytes by visual inspection of the mitochondrial network in confocal microscopy images **(**Supplementary Fig. 4 **)**. After confirming mitochondria-specific staining with MitoTracker Green (MTG) and TMRE **(**Supplementary Fig. 4 **)**, we separately stained astrocytes with the two dyes and analyzed mitochondrial content **(**Supplementary Fig. 4 **)** and Δψm **(**Supplementary Fig. 4 **)** with flow cytometry. While mitochondrial content was normal, the Δψm was decreased in PINK1-deficient astrocytes. Finally, measuring mitochondrial respiration with extracellular flux analysis showed that both basal and maximum respiration were reduced in PINK1-deficient astrocytes compared to WT cells **(**Supplementary Fig. 4 **)**.

### Normal glutamate uptake of PINK1-deficient astrocytes

Astrocytes play an important role in clearing neurotransmitter from the synaptic cleft through active import of glutamate through the glutamate transporter. To study whether PINK1 deficiency affected glutamate uptake, we incubated mixed glia with medium containing 200 μM glutamate and measured its import into astrocytes by determining the remaining glutamate concentration in the medium over time (only astrocytes take up glutamate). There was no difference in the amount or kinetics of glutamate uptake between WT and *PINK1* ^−/−^ astrocytes **(**Supplementary Fig. 5 **)**. Because it had been reported that astrocytic glutamate uptake is inhibited by the mitochondrial and PD-linked toxin MPP^+^^52^, we studied whether PINK1-deficient astrocytes are more sensitive to inhibition of glutamate uptake by MPP^+^ as well as another complex I inhibitor, rotenone. MPP^+^ and rotenone drastically inhibited glutamate uptake in both WT and PINK1-deficient astrocytes **(**Supplementary Fig. 5 **)**. However, glutamate uptake was inhibited to a similar extent in WT and *PINK1* ^−/−^ astrocytes by the complex I inhibitors, except for MPP^+^ at 35 min **(**Supplementary Fig. 5 **)**. Thus, glutamate uptake of PINK1-deficient astrocytes is normal under basal conditions, although there might be a tendency for increased sensitivity to MPP^+^ and possibly rotenone-mediated inhibition of glutamate uptake at early time points.

## Discussion

**Figure 8:**
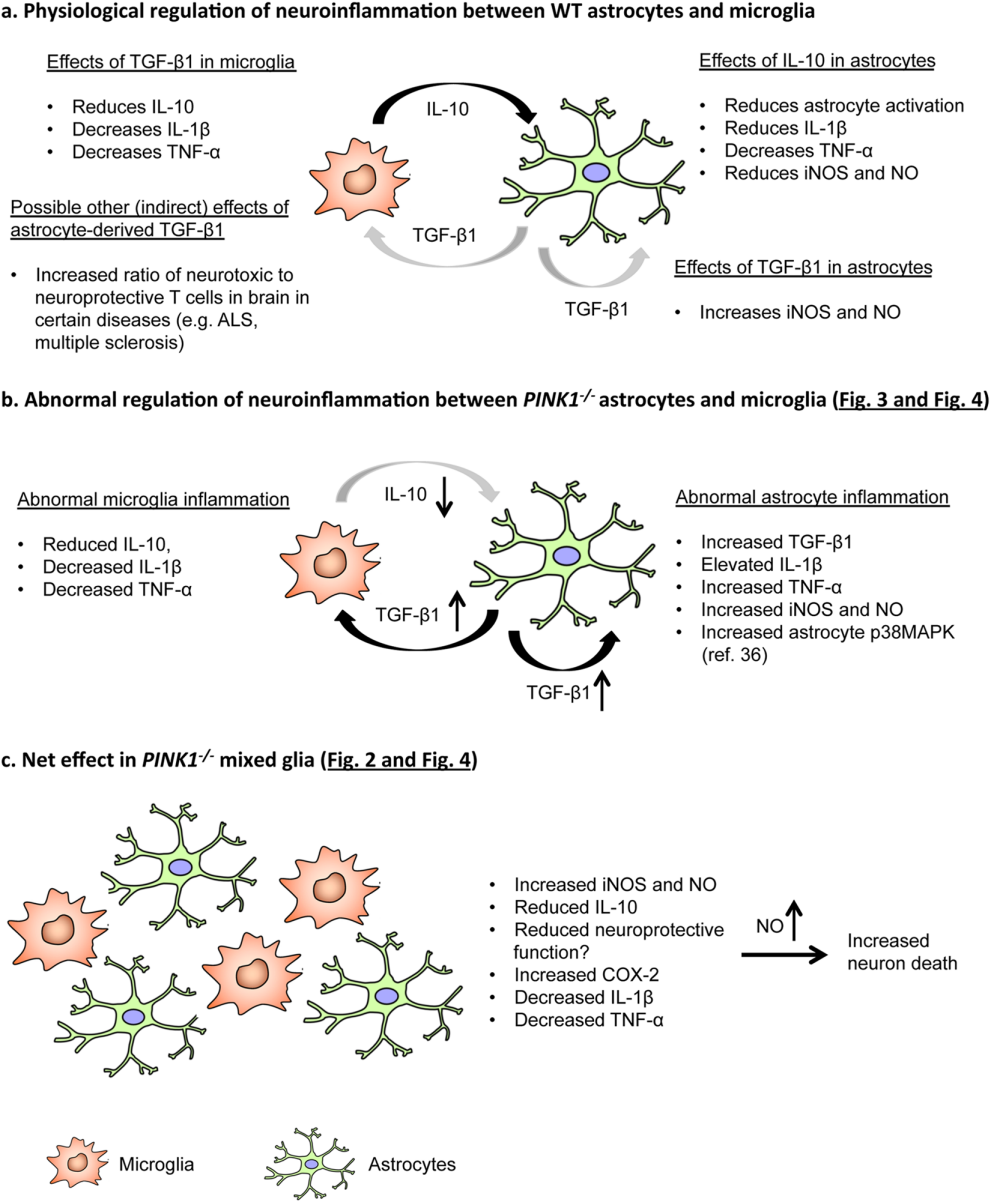
Model for abnormal inflammatory gene expression in astrocytes, microglia and mixed glia cells. (**a**) Reciprocal control of neuroinflammation between microglia and astrocytes by key regulatory cytokines IL-10 and TGF-β1, as described in the Discussion and supported by references cited therein. (**b**) Model illustrating how decreased microglial IL-10 expression and increased astroglial TGF-β1 may result in secondary abnormalities of inflammatory gene expression and NO production in pure *PINK1* ^−/−^ microglia and *PINK1* ^−/−^ astrocytes (Figs 3 and 4). Increased p38MAPK activity in *PINK1* ^−/−^ astrocytes^29^ further supports the gene expression changes measured in this work. (**c**) Net changes of inflammatory gene expression/NO production in mixed astrocytes/microglia (Figs 2 and 4), and model for NO-mediated neuron death. For further details, see Discussion and references cited therein.

Neuroinflammation is an important pathogenic mechanism in idiopathic PD, but its involvement in familial forms of PD is less clear. Recent studies have shown that αS^3–7^ and LRRK2^3,8–11^ promote neuroinflammation. However, studies on how defects in recessive PD genes influence neuroinflammation are few^21,22^. The main goal of this work was to characterize the neuroinflammatory profiles of *PINK1* ^−/−^ glia cells, and to test whether alterations in glia-mediated innate immunity result in increased neuron death, with a special emphasis on mixed glia. Mixed glial cells are most relevant to study neuron death, because neither astrocytes nor microglia exist in isolation in the brain and both glia types reciprocally affect their neuroinflammatory potential, as described below. We show that loss of PINK1 alters LPS/IFN-γ induced neuroinflammation in a glia type-specific manner, increasing pro-inflammatory mediators in astrocytes (TNF-α, IL-1β and NO) and in mixed glia (NO), while attenuating both pro-inflammatory (TNF-α, IL-1β) and anti-inflammatory (IL-10) mechanisms in microglia. Importantly, the finding that IL-10 expression is lower in *PINK1* ^−/−^ microglia provides a mechanistic explanation for the cytokine profile and NO production observed in *PINK1* ^−/−^ astrocytes and mixed glia. As a potent anti-inflammatory cytokine, IL-10 inhibits production of TNF-α and IL-1β, decreases NO biosynthesis and attenuates immunological activation of astrocytes^40,53–55^. Thus, decreased IL-10 expression by *PINK1* ^−/−^ microglia may (in part) be responsible for elevated levels of TNF-α, IL-1β and NO in *PINK1* ^−/−^ astrocytes and mixed glia. Reciprocally, increased expression of TGF-β by astrocytes may down-regulate expression of IL-1β and IL-10 in microglia^55^. On the other hand, lower IL-10 expression by microglia did not cause reduced TGF-β expression in astrocytes^55^. Rather, we find that TGF-β transcript levels are increased in *PINK1* ^−/−^ astrocytes. Because aged astrocytes display increased expression of TGF-β1^56^, we speculate that up-regulation of TGF-β1 may be caused by age-related changes in *PINK1* ^−/−^ astrocytes. Alternatively, compensatory changes in the IL10 signaling pathway may be involved. Independent of the exact mechanism(s), TGF-β1 can exert both anti-inflammatory^41^ and pro-inflammatory^44,45^ actions, depending on context and disease. Importantly, TGF-β1 promotes iNOS expression in astrocytes in response to LPS and IFN-γ^57^, and increased expression of TGF-β in *PINK1* ^−/−^ astrocytes may therefore have contributed to elevated NO production in these cells. Moreover, astrocyte-specific up-regulation of TGF-β1–through diminishing neuroprotective functions of microglia and altering the balance between neurotoxic and neuroprotective T cells^44^ -may enhance inflammation-induced neuronal loss in PD. In contrast to astrocytes, *PINK1* ^−/−^ microglia show reduced TGF-β1 transcript levels at baseline (without inflammation), as well as a tendency for reduced TGF-β1 expression after treatment with LPS/IFN-γ (not statistically significant). Reduced basal expression of TGF-β1 may interfere with the neuroprotective function of *PINK1* ^−/−^ microglia, because up-regulation of TGF-β1 in microglia in response to brain injury and neurodegeneration is considered neuroprotective^58,59^. On the other hand, decreased expression of TNF-α and IL-1β by *PINK1* ^−/−^ microglia shows that PINK1 loss simultaneously attenuates the pro-inflammatory features of microglia. Together with the observation that NO production by LPS/IFN-γ treated *PINK1* ^−/−^ microglia is normal, these results show that lack of PINK1 does not cause an overt change in the direction of microglia-mediated neuroinflammation. Instead, our results suggest that diminished IL-10 production by *PINK1* ^−/−^ microglia indirectly enhances astrocyte-mediated neuroinflammation and neuron death in mixed glia, through mechanisms described above and illustrated in the model depicted in Fig. 8. Indeed, a pro-inflammatory shift (increased NF-κB activity and elevated levels of NO, TNF-α, IL-1β) is evident in pure *PINK1* ^−/−^ astrocytes that are derived from mixed glia. Moreover, astrocytes are the main source of NO production in mixed glia cultures, effectively suppressing NO synthesis by microglia^60^.

Elevated p38MAPK activity has been reported in *PINK1* ^−/−^ astrocytes^29^, suggesting that increased p38MAPK signaling may contribute to augmentation of NO levels in these cells. Moreover, in the brain of mice, astrocyte-specific p38α expression inhibits brain microglia/macrophage inflammation and elevated TNF-α and IL-1β expression after LPS injection^61^. Therefore, increased p38MAPK activity in *PINK1* ^−/−^ astrocytes^29^ may also have played a role in the observed decline of TNF-α and IL-1β expression in *PINK1* ^−/−^ microglia (that were derived from mixed cultures and grown in mixed glia-conditioned medium before stimulation with LPS/IFN-γ). While IL-1β and TNF-α are neurotoxic at high levels, they also stimulate the production of glia-derived neurotrophic factor (GDNF) under inflammatory conditions^62^. Because GDNF exerts potent neurotrophic effects on dopamine neurons^63,64^, impaired LPS/IFN-γ induced IL-1β and TNF-α expression in microglia may render dopamine neurons of *PINK1* ^−/−^ mice more susceptible to mild inflammation-induced degeneration, which is a topic of future investigation. Finally, we note that PINK1 was previously shown to stimulate IL-1β signal transduction via interaction with several proteins that function downstream of the IL-1β receptor^35,36^. Our finding of reduced LPS/IFN-γ-induced IL-1β expression in *PINK1* ^−/−^ microglia is therefore consistent with PINK1’s role to enhance IL-1β signaling.

By co-culturing WT primary cortical neurons with either WT or *PINK1* ^−/−^ mixed glia cells, we established that PINK1 deficiency in glia cells is sufficient to increase LPS/IFN-γ-induced apoptosis of normal neurons. Moreover, we show that conditioned medium from PINK1^−/−^ mixed glia confers increased NO-dependent toxicity to SH-SY5Y neuroblastoma cells, compared to conditioned medium from WT mixed glia. Similar to our findings, loss of DJ-1 in astrocyte-enriched cultures rendered co-cultured neurons more vulnerable to LPS-induced death, which was attributed to increased iNOS expression downstream of p38MAPK signaling^22^. In addition, it was reported that acutely prepared brain slices from PINK1-deficient mice produce higher levels of TNF-α, IL-1β and IL-6^34^. However, acute brain slicing is more akin to brain injury than inflammation as tested in our study, and it is therefore not possible to directly compare the results of these two works. Finally, aged PINK1-deficient mice were reported to develop spontaneous but subtle increases of GFAP and Iba-1 reactivity in certain brain areas^65^.

To the best of our knowledge, we show for the first time that deletion of a recessive PD gene (i.e., PINK1) increases αS-induced NO production in mixed glia, pure astrocytes and pure microglia. The majority of αS in our studies was soluble and migrated as a monomer on the SDS polyacrylamide gel, although we cannot exclude that a small amount of oligomers were formed spontaneously. It has been shown that soluble oligomers of αS are most neurotoxic when added to microglia^14^. However, monomeric αS was taken up with equal efficiency, and stimulated TNF-α and IL-6 expression with comparable or higher efficiency, than fibrillar αS in microglia in another study^12^. Monomeric αS also promoted nuclear translocation of NF-κB in microglia and was taken up by aged astrocytes, where it promoted inflammatory cytokine production with equal potency to fibrillar αS^12^. Other groups independently demonstrated that monomeric αS preparations are able to induce inflammatory gene expression in microglia^66,67^ and astrocytes^13^. Because αS is abundantly expressed in the brain, loss of PINK1 may enhance αS-induced, glia-dependent neuroinflammation. Interestingly, dopamine neuron loss induced by viral overexpression of αS in the substantia nigra is exacerbated in PINK1-deficient mice^46^, and A53T-αS transgenic mice lacking PINK1 have more severe phenotypes than either mutant mouse line alone^47^. Our results indicate that increased NO production may have been partly responsible for the aggravated neurotoxicity of αS in these studies with *PINK1* ^−/−^ mice. NO promotes nitration and aggregation of αS^68,69^, potentially explaining the increase of serine 129-phosphorylated αS inclusions in the brain and spinal cord of *PINK1* ^−/−^ mice overexpressing αS^46,47^.

Early studies showed that loss of PINK1 in neurons and fibroblasts is associated with increased cytosolic and/or mitochondrial ROS, as well as decreased Δψm and mitochondrial deficits^25–28^. A minor goal of our work **(**Supplementary Figs 3–5 **)** was to characterize ROS levels, mitochondrial bioenergetics and glutamate uptake in *PINK1* ^−/−^ astrocytes at basal levels (without inflammation). We confirm increased cellular ROS and reduced Δψm reported in *PINK1* ^−/−^ astrocytes in a previous study^29^. However, in contrast to this study^29^ we find no reduction of mitochondrial mass in astrocytes lacking PINK1. We show newly that-despite increased ROS levels and reduced mitochondrial respiration-glutamate uptake of *PINK1* ^−/−^ astrocytes is normal. In addition, the vulnerability of *PINK1* ^−/−^ astrocytes to mitochondrial complex I inhibitor-induced impairments of glutamate uptake is comparable to WT. Therefore, oxidative stress and decreased mitochondrial respiration in non-inflamed *PINK1* ^−/−^ astrocytes do not affect glutamate uptake. *PINK1* ^−/−^ astrocytes also showed normal gross mitochondrial morphology based on confocal microscopy, although we cannot exclude more subtle defects in form factor or branching. We note that the presence and magnitude of mitochondrial defects in PINK1 deficiency are cell type-dependent^31,33^, and no gross morphological deficits of mitochondria were observed in the brain of PINK1-deficient mice^30^. However, it has recently been shown that inflammation results in transient mitochondrial fragmentation in astrocytes that is partially reversed by induction of autophagy^70^. Thus, it will be interesting in the future to study whether PINK1 loss affects the vulnerability to inflammation-induced mitochondrial fission and, if so, whether this might be causally linked to altered neuroinflammation in astrocytes.

In summary, we reveal enhanced glia-mediated neuroinflammation, and in particular NO, as a new potential pathogenic pathway in PINK1-related familial PD. Collectively, our results suggest a (literature-supported) model, where loss of PINK1 results in abnormalities of microglial IL-10 expression and astroglial TGF-β expression, which in turn affects the neuroinflammation phenotype of the other glia type in a reciprocal manner **(**Fig. 8 **)**. We propose that impairments of IL-10 production by PINK1^−/−^ microglia leads to increased synthesis of pro-inflammatory mediators in astrocytes and mixed glia. We also show for the first time that PINK1 deficiency (and lack of any recessive PD gene) increases soluble αS-induced NO production in astrocytes, microglia and mixed glia. This suggests that lack of PINK1 may predispose to αS-induced neuroinflammation, and provides important new insights into possible mechanisms underlying the recently reported increased vulnerability of *PINK1* ^−/−^ mice to αS-induced dopamine neuron loss.

## Materials and Methods

### Animals

*PINK1* ^−/−^ mice have been described previously^71^ and were backcrossed for at least 15 generations onto the pure C57BL/6 J background. Animal experiments were performed according to the “Guide for the Care and Use of Laboratory Animals”, Eight Edition, 2011 (The National Academic Press, Washington, D.C.) and the “National Regulations of China for the Care and Use of Laboratory Animals” and approved under Heilongjiang province permit SYXK-2016–007 of Harbin Medical University.

### Antibodies, oligonucleotides and tissue culture media

Oligonucleotides, antibodies and antibody dilutions are listed in Supplementary Table 1. Tissue culture media, B-27 supplement, trypsin, soybean trypsin inhibitor and Hanks balanced salt solution (HBSS) were from Life Technologies. Fetal bovine serum (FBS) was from JYK Biotechnology, China.

### Cultures of mixed glia, pure astrocytes and pure microglia

All types of glia were cultured in DMEM/F12 containing 10% FCS (medium). To establish mixed astrocyte/microglia cultures, primary cortical cells from neonatal mouse brains were isolated as described^72^ but expanded without shaking. After trituration and centrifugation (5 min at 300 *g*), cells isolated from individual brains were plated into 21 cm^2^ (6-cm) poly-L-lysine coated (Sigma P6282, 50 μg/ml) plates. Medium was changed every 2–3 days and on DIV 8–10, when cells reached confluence, they were trypsinized and passaged 1:2. Five days later, cells were plated at 2 × 10^5^ cells/cm^2^ into 12-well or 24-well plates. Mixed glia contained ~81–85% astrocytes and ~14–17% microglia. Pure astrocytes were obtained from DIV 8–10 mixed glia monolayers by removal of microglia after treatment with 10 μM cytosine β-D-arabinofuranoside (AraC, Sigma c6645) for 3–5 days, followed by a one-hour incubation with 75 mM L-leucine methyl ester (Sigma L1002, adjusted to pH 7.4)^73^. Astrocytes were washed twice with medium and allowed to recover for 24–30 hours before being plated for experiments (2 × 10^5^ cells/cm^2^). Mild trypsinization was used to establish microglia-enriched cultures^74^. Briefly, DIV11–15 mixed glia cells were incubated with a 3-fold dilution of 0.25% trypsin-EDTA (in serum-free DMEM/F12) for about 30 min, resulting in detachment of the entire astrocytes layer. Remaining attached microglia were washed twice with medium and cultured for 3–5 days with mixed glia-conditioned medium. Microglia were dissociated with 0.25% trypsin-EDTA and re-plated for experiments at 5–8 × 10^4^ cells/cm^2^.

### Measurement of reactive oxygen species in retrovirus-transduced primary astrocytes

Coding sequences for cytosolic and mitochondria-targeted roGFP^75^ were amplified by PCR from Addgene plasmids #49435 and #49437. PCR products were sub-cloned into a derivative of the retroviral vector MFG^76^. 10 μg of the resulting plasmids pSFG-Cyto-roGFP and pSFG-Matrix-roGFP were transfected into PlatE packaging cells^77^. High-titer recombinant retroviruses in medium (~5 × 10^7^ transducing units/ml) were collected 48 and 72 hours after transfection, sterile-filtered (0.45 μm) and frozen at −80 °C. Astrocytes were infected with recombinant retrovirus in presence of 4 μg/ml polybrene (Sigma H9268), resulting in >90% of cells being transduced 2–3 days later. ROS in cytoplasm and mitochondrial matrix were measured in stably transduced astrocytes, by exciting the cells at 405 nm (oxidized roGFP) and 488 nm (normal roGFP) and taking images of the emitted fluorescence in the confocal microscope. Fluorescence emission was quantified with Image J software, and the ratio of emitted fluorescence (F405/F488) was calculated as a measure of ROS in individual cells. An increased ratiometric fluorescence (F405/F488) indicates heightened oxidative stress in the compartment in which roGFP is expressed.

### Measurement of mitochondrial content, membrane potential (Δψm) and respiration

Mitochondrial content and Δψm were analyzed separately by flow cytometry in primary astrocytes after incubation with 100 nM MitoTracker Green (Cell Signaling 9074) for 15 min and 100 nM TMRE (Sigma 87917) for 15 min at 37 °C, respectively. Mitochondrial oxygen consumption rate (OCR) of astrocytes was measured with the XF^e^24 Extracellular Flux Analyzer (Seahorse Bioscience). 5 × 10^4^ cells/well were plated into poly-L-lysine coated XF^e^24 plates and incubated overnight at 37 °C, 5% CO_2_. The next day, medium was changed to XF assay medium (Seahorse Bioscience) containing 25 mM glucose and 1 mM sodium pyruvate and cells were incubated at 37 °C in a non-CO_2_ incubator for 1 hour for calibration. Basal respiration and respiration after addition of 1 μM oligomycin, 1 μM FCCP and 1 μM rotenone/antimycin (each) were analyzed sequentially with three measurements for each phase of the respiration profile. OCR was normalized to protein concentration in each well determined in whole-cell lysates using the Pierce BCA kit.

### Purification of recombinant mouse αS and measurement of LPS in purified αS

Mouse αS CDS was amplified by RT-PCR using gene-specific primers **(**Supplementary Table 1) and sub-cloned into the bacterial expression vector pET-21a. The correct αS CDS was confirmed by sequencing. Pellets from *E*. *coli BL21* (DE3) bacteria transformed with the resulting pET-21a-mouse αS plasmid were resuspended in high-salt buffer (1 M NaCl, 25 mM Tris, pH 7.4, 1 mM EDTA) containing PMSF, subjected to sonication, heated to 100 °C for 10 min and centrifuged at 15,000 *g* for 30 min. The supernatant was dialyzed overnight against a 100-fold volume of buffer (25 mM Tris, pH 7.4). The dialyzed sample was ultra-centrifuged at 200,000 *g* for 15 min, the supernatant was applied to a Resource Q column (GE Healthcare) and fractions were eluted with a 0–0.5 M NaCl gradient. Pooled fractions A10 and A11 containing pure, monomeric αS as judged by inspection of the SDS gel (Fig. 5A) were used in experiments. LPS in serial dilutions of the protein was measured with a competitive ELISA assay (Elabscience, E-EL-0025) and quantified using an internal LPS standard curve.

### Quantification of LPS/IFN-γ-induced and αS-induced NO production in glia

Cells were serum-starved for 24 hours in FBS-free medium (mixed glia and astrocytes) or medium containing 2% FBS (microglia). Subsequently, cells were incubated for 24 hours in DMEM/F12–2% FBS with different concentrations of LPS (Sigma L2880) or recombinant αS (as indicated in figures) in presence of 10 ng/ml IFN-γ (Cell Signaling 5222-SC). NO levels in medium were measured indirectly via quantification of NO-derived nitrite (NO_2_^-^) using the Griess reagent assay^78^. Briefly, the collected medium was mixed with an equal volume of 1 × Griess reagent (Sigma G4410), incubated for 15 min at RT in the dark, and the absorption at 540 nm was measured immediately. Nitrite concentrations were determined using a nitrite standard and normalized to protein content of the same well (measured with the Pierce BCA assay). In inhibitor studies, the p38MAPK inhibitor (SB203580), broad-spectrum JNK inhibitor (SP600125) and pan-JAK (Janus kinase) inhibitor were used at 30 μM, 20 μM and 30 μM, respectively. Inhibitors were present from one hour prior to until the end of the 24-h LPS/IFN-γ treatment.

### Quantification of inflammatory enzyme and cytokine expression by real-time PCR

Cellular RNA was isolated with Trizol reagent, and first strand cDNA was synthesized with the Prime Script RT kit (Takara Inc.) from 500 ng total RNA of each sample. Two μl of the resulting cDNA (5-fold dilution) was subjected to real-time PCR using SYBR Premix Ex Taq II (Tli RNase H Plus) master mix (Takara Inc). Forward and reverse PCR primers are listed in Supplementary Table 1 and were in different exons to avoid amplification of genomic DNA. Melting curve analysis was done to confirm single PCR products. We used the 2^−ΔCt^ method^79^ to calculate mRNA expression of each gene relative to β-actin after initial confirmation that neither loss of PINK1 nor treatment with LPS/IFN-γ altered the expression of the internal standard β-actin (p > 0.05, t-test).

### Western blots

Primary cells were lysed and brain tissue was homogenized with modified RIPA buffer (50 mM Tris-HCl, pH 8.0, 1% Triton X-100, 0.1% SDS, 0.14 M NaCl, 1 mM EDTA, and 1 mM EGTA) containing 1% (v/v) protease inhibitor cocktail (Amresco M250). 20–30 μg total proteins in the cleared lysates (supernatants of 10 min, 12,000x*g* centrifugation) were analyzed by standard Western blot procedures. Anti-GFAP and anti-β-actin antibodies were used at 4 °C overnight, followed by IR-Dye 680RD or IR-Dye 800CW secondary antibodies for 1 hour at room temperature. Bands were visualized using the Odyssey Infrared Imaging System and quantified with ImageJ software.

### Apoptosis of primary neurons co-cultured with mixed astrocytes/microglia

Primary cortical neurons were isolated from newborn (<24-hours old) mice as described^80^. Briefly, dissected cortices were washed in HBSS (pH 7.4) containing 1 g/liter D-glucose and digested in 0.25% trypsin at 37 °C for 15 min. After addition of 0.014% soybean trypsin inhibitor, tissue was gently triturated in HBSS to generate a suspension of mostly single cells, which was collected by centrifugation and resuspended in neuron growth medium (Neurobasal, 2% B-27 supplement, 0.5 mM L-glutamine, 100 U/ml penicillin, 100 μg/ml streptomycin). 1.5 × 10^5^ neurons/well were added to 15-day old primary mixed glia (3.75 × 10^5^/well in poly-L-lysine-coated 24-well plates), whose medium was replaced 24 hours earlier with neural growth medium. Half of the medium was changed 24 hours later and subsequently every three days. Six days after plating neurons, the cultures were treated with 1 μg/ml LPS and 10 ng/ml IFN-γ by replacing half of the medium with fresh LSP/IFN-γ-containing medium, while control cells received 50% fresh medium only. Twenty-four hours later the cells were fixed with 4% paraformaldehyde and permeated with 0.25% Triton X-100 for immunocytochemistry (ICC). ICC was performed using standard buffers with mouse anti-tubulin-βIII antibody (neurons) and rabbit anti-active caspase-3 antibody (apoptosis), followed by washing and incubation with species-specific fluorescent secondary antibodies. Confocal images were taken, and the total number of neurons (tubulin-βIII positive cells) and number of neurons containing active caspase-3 (tubulin-βIII/active caspase-3 double-positive cells) were counted using Image J software to calculate the percentage of apoptotic neurons.

### Survival of SH-SY5Y neuroblastoma cells exposed to glia-conditioned medium

3.8 × 10^5^ mixed glia or pure astrocytes (per well of a 24-well plate) were serum-starved for 24 hours, then treated in serum-reduced (2% FBS) medium with LPS/IFN-γ, LPS/IFN-γ plus 10 μM of the iNOS inhibitor 1400 W in DMSO, or left untreated (DMSO control). Twenty-four hours later, conditioned medium was collected and added to SH-SY5Y neuroblastoma cells (2.4 × 10^4^/well of a 96-well plate; ATCC CRL-2266) that were plated one day earlier. Forty-eight hours later, SH-SY5Y cell viability (survival) was measured with the CCK8 cell counting kit (Dojindo Inc.) according to the manufacturer’s instructions. For each type of conditioned medium above, survival was calculated and expressed as percentage of survival of SH-SY5Y cells exposed to regular (10% FBS) SH-SY5Y neuron growth medium.

### Analysis of NF-κB pathway activity (nuclear p65)

Cultures of primary mixed glia (75–79% astrocytes) were starved for 24 h in serum-free medium and then incubated in medium containing 2% fetal bovine serum, 1 μg/ml LPS and 10 ng/ml IFN-γ. At 10 min, 20 min and 30 min of incubation, cells were fixed in 4% (w/v) paraformaldehyde in PBS and permeated with 0.25% (v/v) Triton X-100. Detection of NF-κB p65 subunit was carried out with standard immunocytochemistry methods using anti-NF-κB p65 antibody and Alexa488-conjugated secondary antibody. DAPI staining was used to label nuclei, and a total of 400–1000 cells were analyzed in three random images of each culture/mouse. The percentage of cells with p65 in the nucleus was calculated as the number of p65/DAPI double-positive nuclei divided by the number DAPI-positive (total) cells and multiplied by 100, and is a measure of NF-κB transcriptional activity.

### Glutamate uptake assays (±mitochondrial inhibitors)

Cells were washed with HBSS and then incubated with HBSS/5 mM glucose containing 200 μM glutamate (Sigma G5889) (t = 0 min, baseline glutamate concentration). The glutamate concentration in HBSS was measured at various times thereafter (up to 105 min) with a glutamate quantification assay and internal glutamate standard according to the manufacturer’s instructions (BioVision, #K629–100). For experiments including mitochondrial inhibitors, 25 μM MPP^+^ (Sigma D048) or 5 μM rotenone (Sigma R8875) was added to astrocyte medium five hours before starting the glutamate uptake assay and was also present in HBSS/5 mM glucose during the uptake assay. Glutamate uptake was normalized to protein concentration (determined with Pierce BCA kit) in each culture/well.

### Statistical analysis

The software Prism 7 (Graph Pad) was used for all statistical analyses. Data are presented as mean ± SD unless stated otherwise for the indicated number of mice/samples (n). Cytokine expression, nuclear p65 translocation and glutamate uptake were analyzed with two-way ANOVA and Bonferroni’s multiple comparisons test. NO production in presence of signaling inhibitors (p38MAPK, JNK, JAK) and in response to αS was analyzed with two-way ANOVA and Tukey’s multiple comparisons test. Primary cortical neuron apoptosis and SH-SY5Y neuroblastoma cell survival were analyzed with ANOVA and Tukey’s multiple comparisons test. Other results were analyzed by unpaired, two-tailed t-test, except when the F-test revealed unequal variances of two populations. In this case, an unpaired t-test with Welch’s correction was performed. Differences were considered statistically significant at *p < 0.05, **p < 0.01 ***p < 0.001 and ****p < 0.0001.

### Availability of data

The data used or analyzed during the current study are available from the corresponding author on reasonable request.

## Electronic supplementary material

Supplementary Figures and Tables
