# Supplementary figures and images for: Lack of PINK1 alters glia innate immune responses and enhances inflammation-induced, nitric oxide-mediated neuron death
Source: Sci Rep. 2018 Jan 10;8:383. doi: 10.1038/s41598-017-18786-w (PMC5762685; doi:10.1038/s41598-017-18786-w)

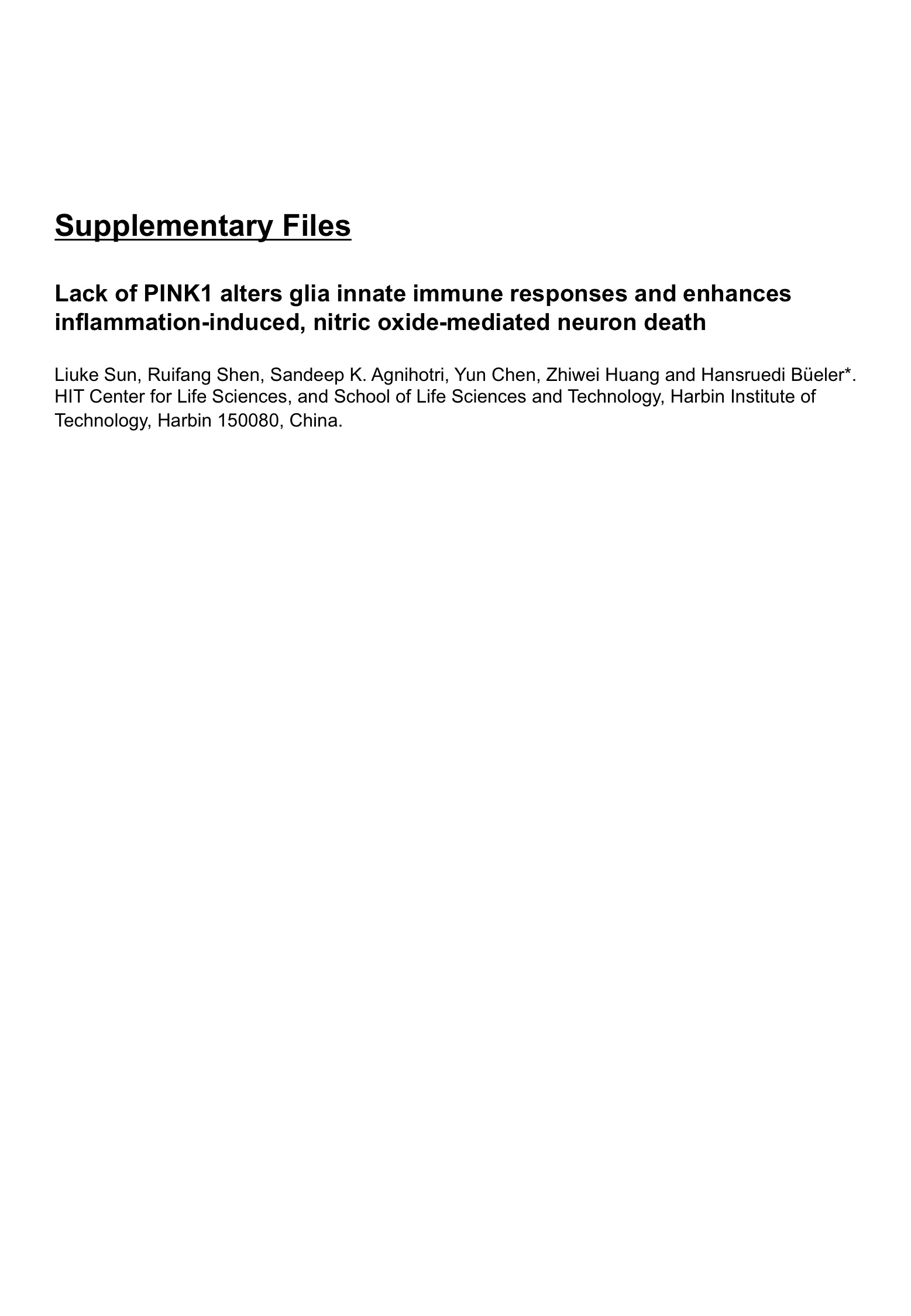


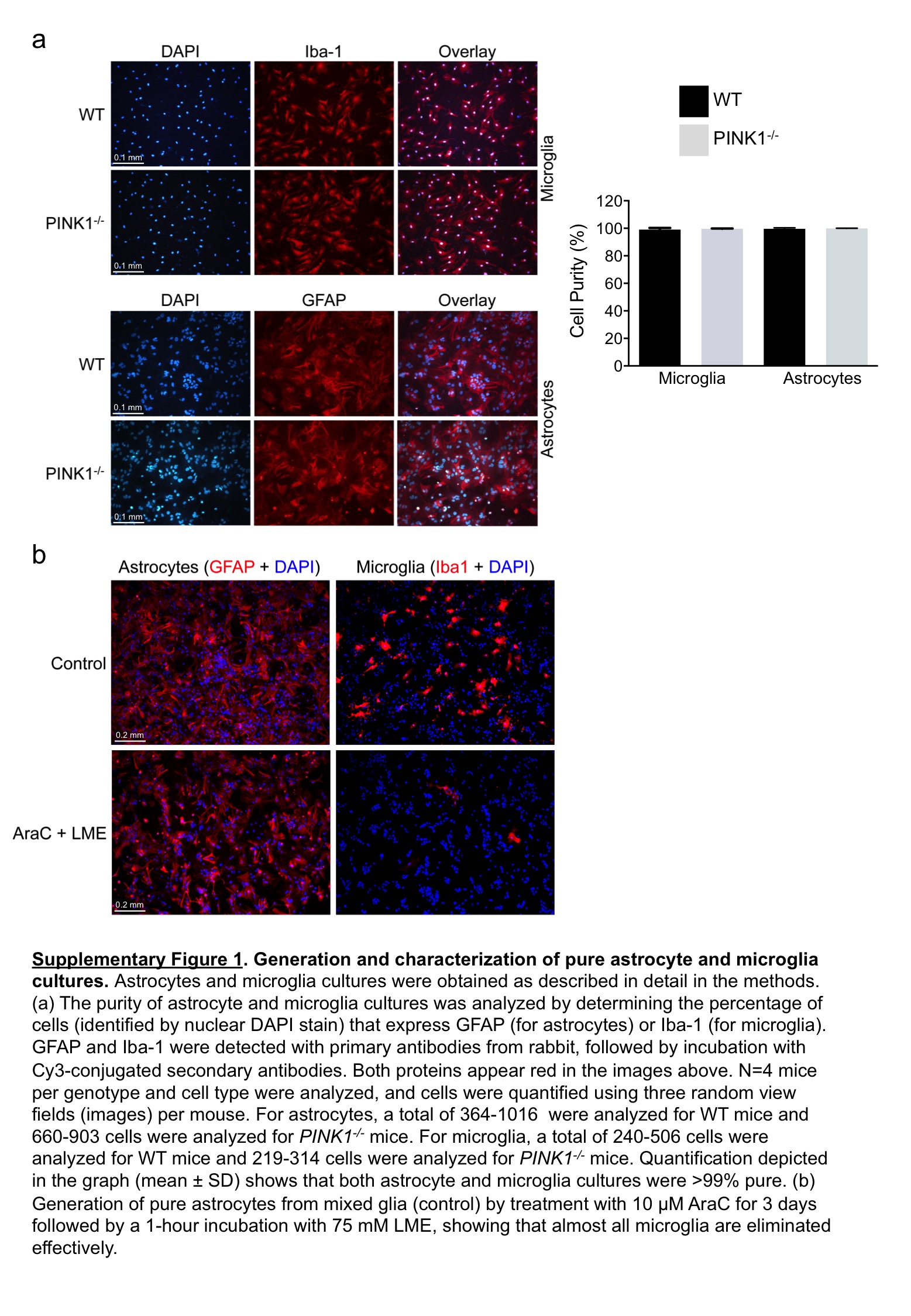


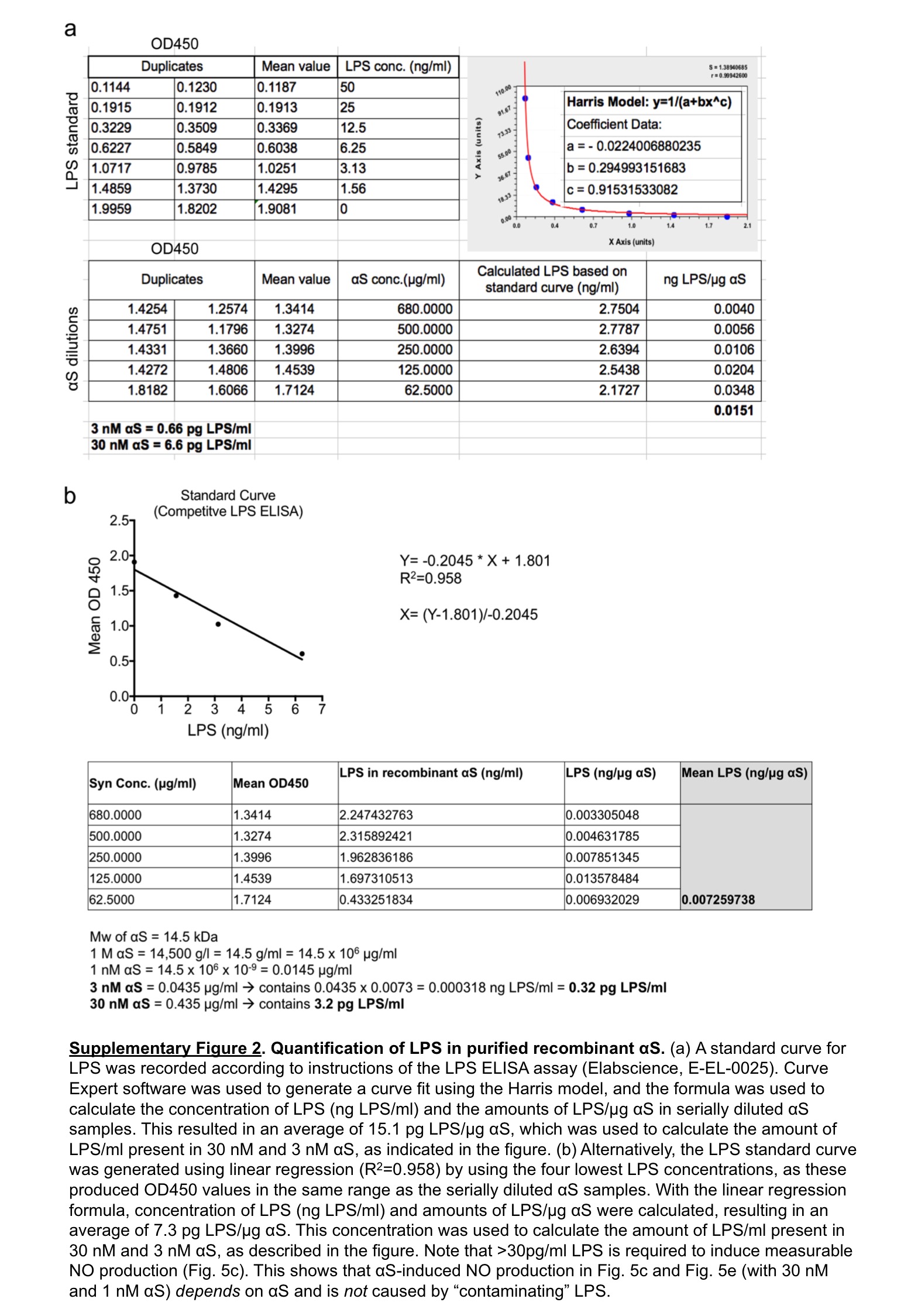


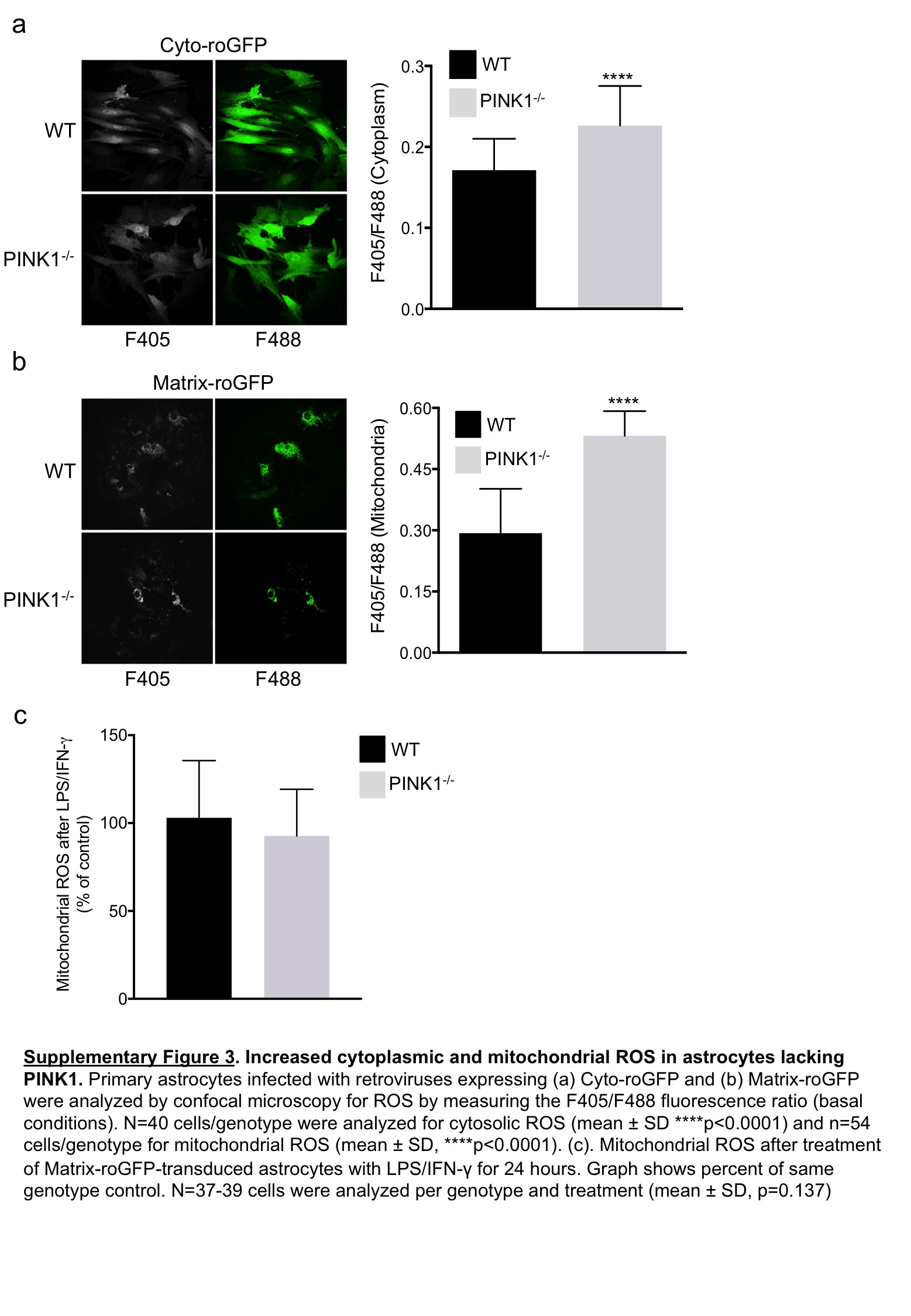


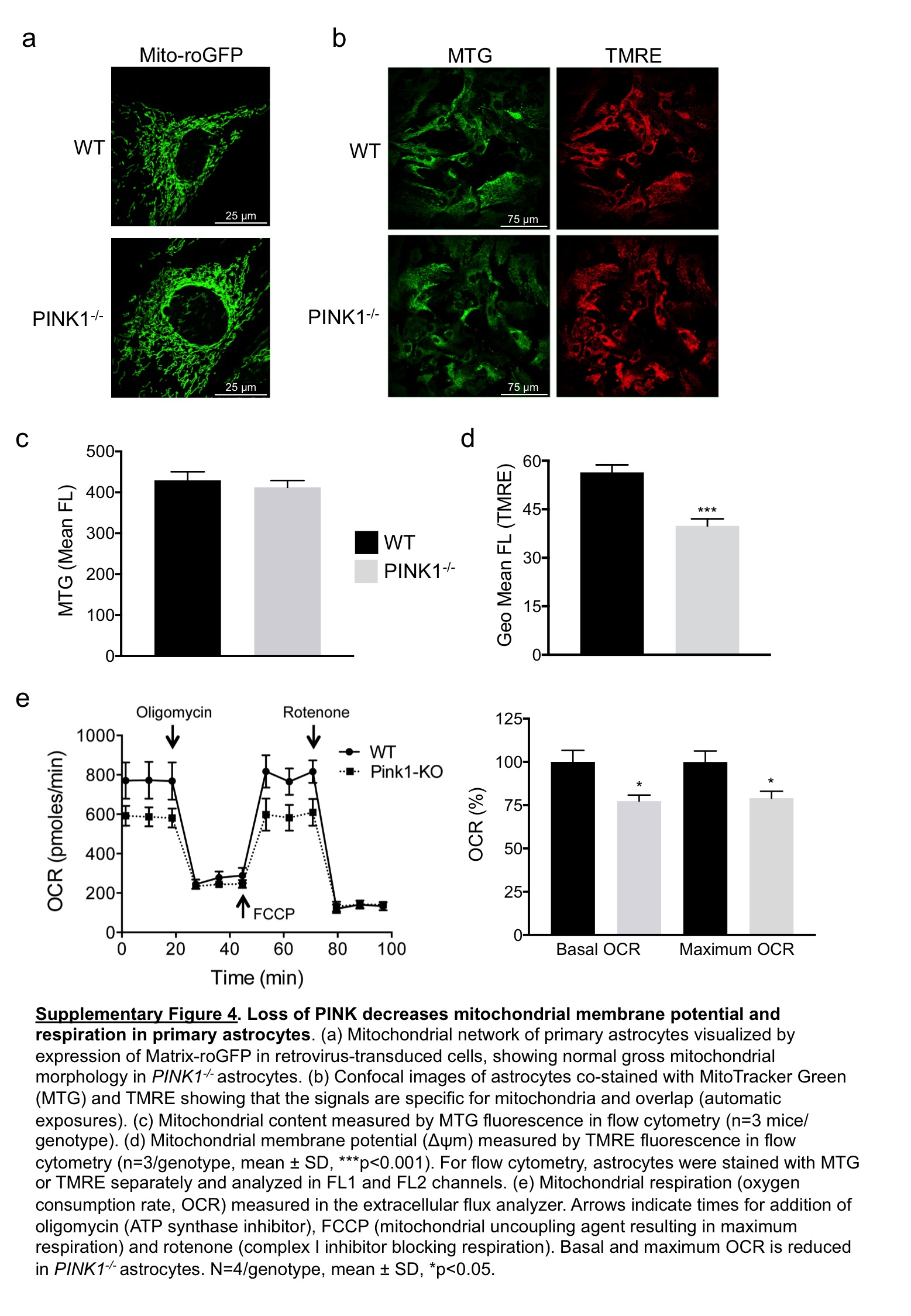


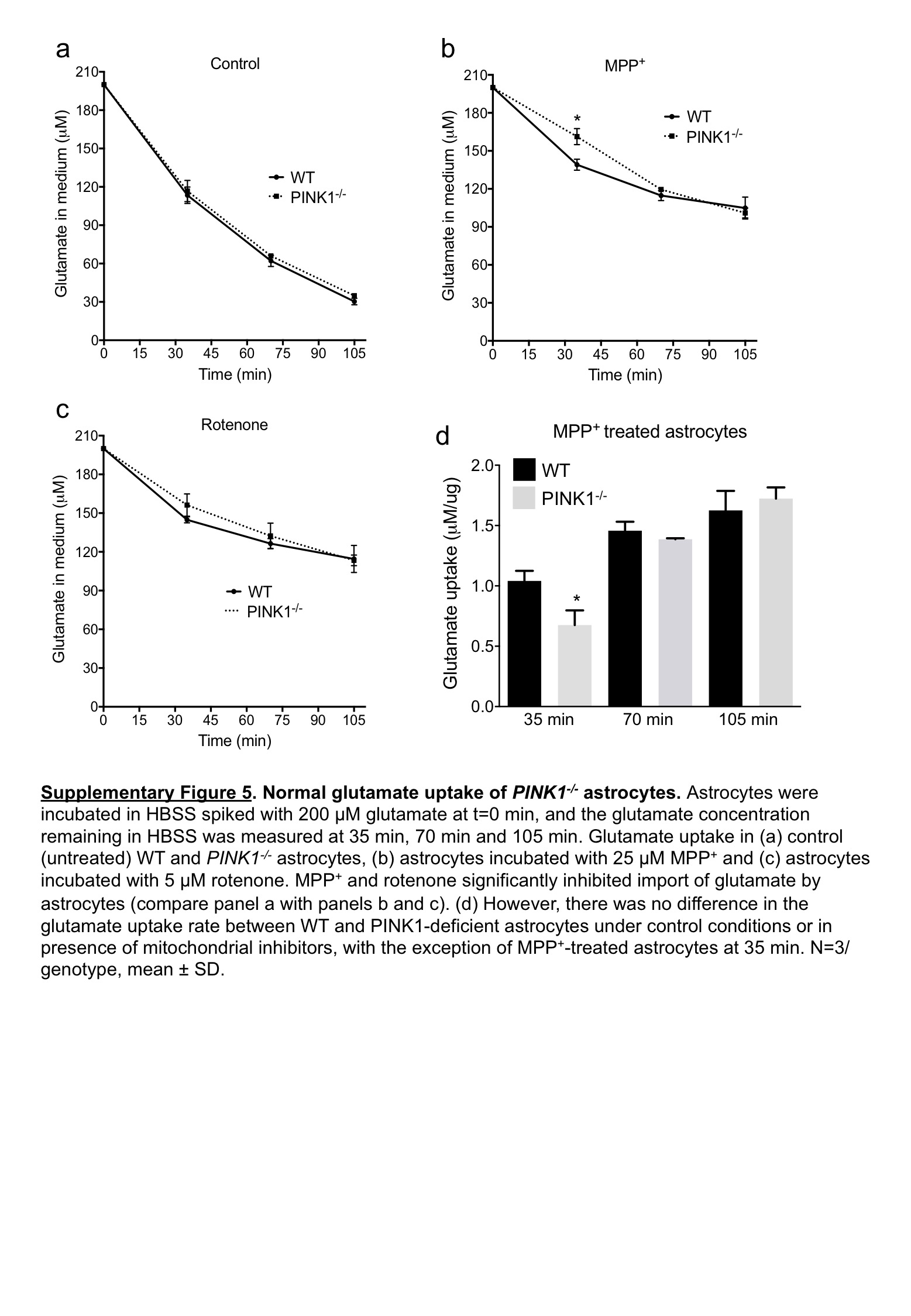


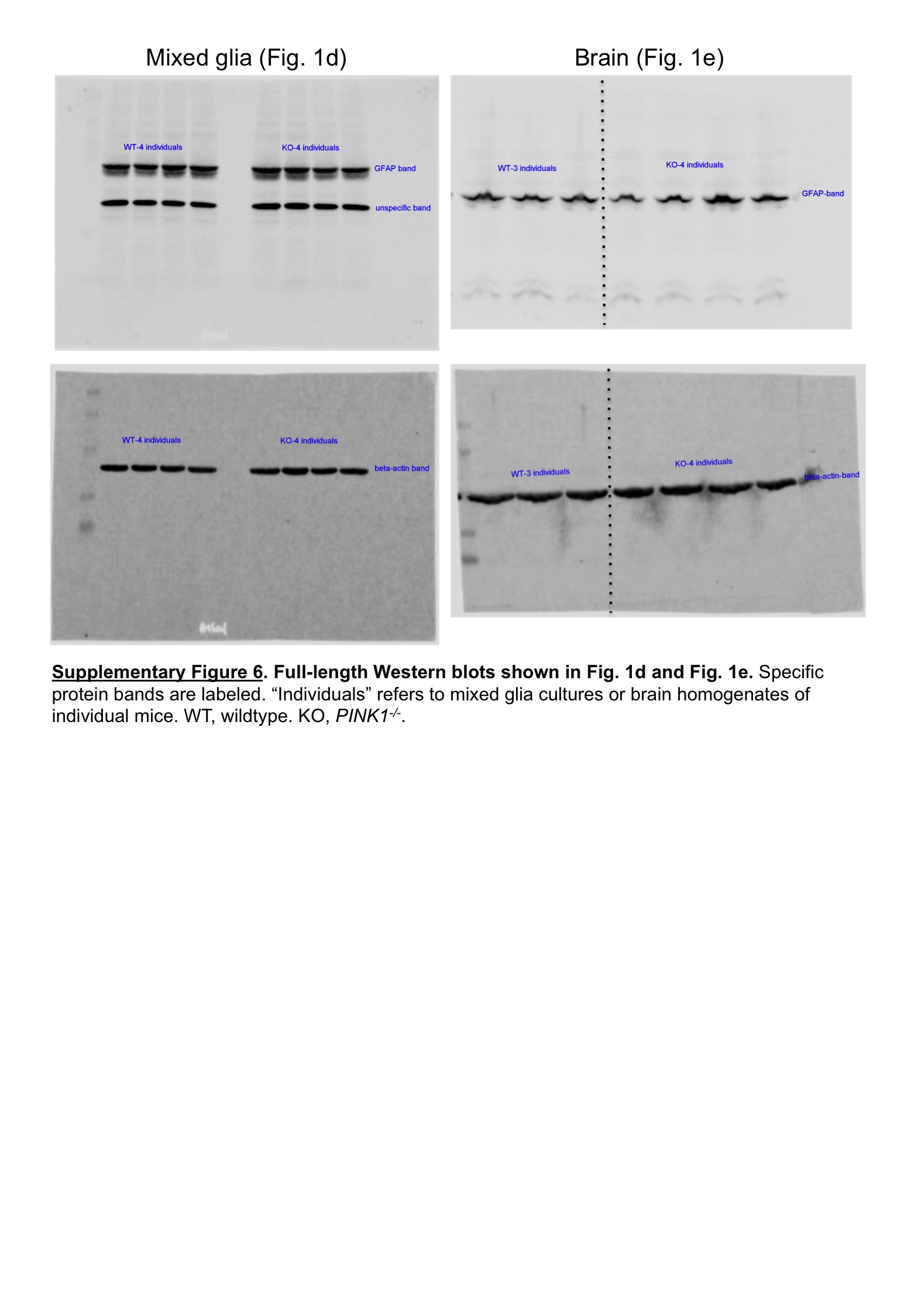


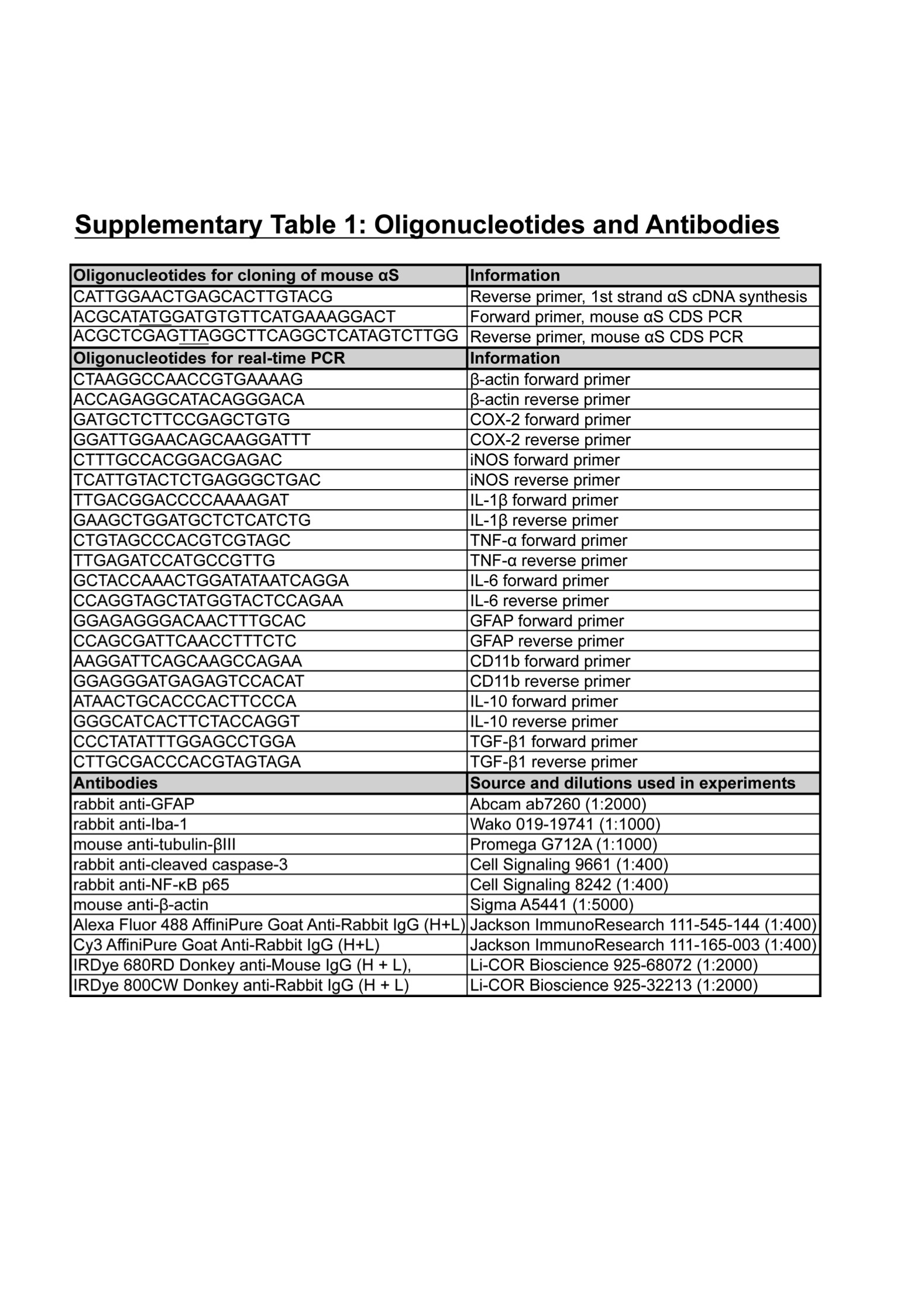

Supplement: Supplementary file 1 — Supplementary Figures and Tables [file 41598_2017_18786_MOESM1_ESM.docx]
